# Multi-Strategy Improved POA for Global Optimization Problems and 3D UAV Path Planning

**DOI:** 10.3390/biomimetics10110760

**Published:** 2025-11-11

**Authors:** Rui Zhang, Jingbo Zhan, Jianfeng Wang

**Affiliations:** 1 School of Engineering Science, Shandong Xiehe University, Jinan 250107, China; zhangrui@sdxiehe.edu.cn; 2 Airforce Aviation Repair Institute of Technology, National University of Defense Technology, Changsha 410073, China; 3 School of Computer and Control Engineering, Northeast Forestry University, Harbin 150040, China; jingbo.zhan@nefu.edu.cn; 4 South Korea College of Design, Hanyang University, Ansan 15588, Gyeonggi-do, Republic of Korea

**Keywords:** 3D UAV path planning, intelligent optimization algorithm, pelican optimization algorithm, IEEE CEC2017

## Abstract

With the rapid development of smart manufacturing and the low-altitude economy, drone technology—as a vital component of next-generation intelligent equipment—has garnered significant attention from researchers. Path planning, one of the core challenges in drone technology advancement, directly impacts the efficiency and safety of drone mission execution. However, most existing drone path planning algorithms suffer from issues such as requiring extensive interactive information or being prone to getting stuck in local optima. This study introduces a multi-strategy enhanced Pelican Optimization Algorithm (MIPOA) tailored for UAV path planning. To improve the quality of the initial population, a hybrid initialization approach combining low-discrepancy sequences with heuristic refinement is developed. The low-discrepancy component promotes a more uniform distribution across the search space, while the heuristic mechanism enhances the fitness of selected individuals and reduces redundant exploration. Furthermore, a subgroup mean-guided updating strategy is designed to accelerate convergence toward the global optimum. To maintain exploration ability, a random reinitialization boundary mechanism is incorporated, effectively preventing premature convergence. To validate the algorithm’s performance, MIPOA is compared with eleven benchmark metaheuristics on the CEC2017 test suite, and statistical analyses confirm its superior optimization capability. Finally, MIPOA is applied to 3D UAV path planning under four threat scenarios in a realistic environment, demonstrating robust adaptability and achieving successful mission completion.

## 1. Introduction

In today’s era of rapid technological advancement, the continuous progress and widespread application of drone technology are profoundly transforming the operational models of numerous industries, with its importance becoming increasingly prominent. In agriculture, the application of drones across various scenarios—including irrigation, fertilization, pesticide application, weed management, plant growth monitoring, crop disease management, and field phenotyping—can effectively enhance efficiency in smart agriculture [1]. In logistics distribution, drones can overcome terrain limitations to enable rapid delivery of goods in remote areas or special scenarios [2]. Logistics drones developed by companies like SF Express have established regular delivery networks in certain regions, handling a considerable daily volume of parcels. Delivery timeliness in special areas has improved significantly, effectively driving the development of the logistics industry. In emergency rescue scenarios, their role is irreplaceable. During the critical rescue window following a disaster, drones can rapidly establish emergency communication relay networks to ensure connectivity for affected areas [3]. Drones equipped with thermal imaging modules can penetrate thick smoke to precisely locate trapped individuals and deliver supplies with pinpoint accuracy. In summary, drone technology plays an immeasurable role in agriculture, smart cities, emergency rescue, and numerous other fields. Its in-depth research is an inevitable choice that aligns with the demands of the times, enhances national comprehensive strength, and improves the quality of life for the public. It holds profound practical significance and strategic value.

Within the drone technology ecosystem, path planning technology stands as the undisputed core and cornerstone, determining whether drones can safely, efficiently, and precisely accomplish their missions. Whether in civilian applications like agricultural crop protection [4] and logistics delivery [5], or in specialized domains such as emergency rescue [6] and national defense security, all drone functionalities hinge on the premise of “rationally planning flight paths.” Without scientific path planning, even drones equipped with the most advanced sensors, propulsion systems, and control modules may fail missions due to chaotic flight routes—or worse, trigger safety incidents. Path planning technology for drones serves as the “central nervous system,” coordinating all the capabilities of the unmanned aerial vehicle. Without this core technological support, drones would become like “headless flies.” Consequently, many researchers are studying path planning for drones in three-dimensional space.

Within the drone path planning technology framework, algorithms serve as the core driving force for achieving “safe, efficient, and precise” flight path design. Among these, reinforcement learning algorithms and intelligent optimization algorithms represent the two most widely applied and thoroughly researched mainstream technical approaches, collectively supporting path planning requirements across diverse scenarios. Reinforcement learning algorithms operate on the core principle of “trial-and-error learning.” By enabling the intelligent agent to continuously interact with complex environments, they optimize decision-making strategies through a “reward-punishment” mechanism to derive drone flight trajectories. For example, in 2024, Zhang et al. proposed an improved deep reinforcement learning method for drone path planning in dynamic scenarios by modeling the path planning problem using a Markov decision process [7]. Experimental results demonstrated that the proposed algorithm can accomplish drone path planning tasks in dynamic environments, outperforming classical methods such as A*, RRT, and DQN in terms of planning effectiveness. Zhao et al. proposed a Dual DQN State Split Q-Network (DDQN-SSQN) algorithm that integrates state splitting and optimal states to address path planning for UAV base stations during search missions [8]. This approach enables optimal path planning for UAVs based on deep reinforcement learning (DDQN). Compared to traditional algorithms, the proposed solution achieves faster optimal path planning while demonstrating superior stability and convergence speed. In a dynamic environment with potential threats, Yan et al. examined the survivability probability of UAVs under enemy radar detection and missile attacks [9]. They developed a situational assessment model and proposed a deep reinforcement learning (DRL)-based UAV path planning method utilizing global situational information. This approach demonstrated favorable performance in both static and dynamic mission settings. Although reinforcement learning algorithms demonstrate advantages in autonomous decision-making for path planning in dynamic drone environments, practical applications still face challenges such as strong dependence on environmental modeling, weak generalization capabilities, and high decision complexity when handling multi-constraint, multi-objective tasks. Consequently, many researchers are exploring intelligent optimization algorithms as potential alternative solutions.

Intelligent optimization algorithms are designed based on the principle of “emulating natural laws,” encompassing classic methods such as the Particle Swarm Optimization (PSO) [10], Genetic Algorithm (GA) [11], and Ant Colony Optimization (ACO) [12]. By simulating biological group collaboration and natural selection processes, these algorithms efficiently search for optimal solutions within vast path solution spaces. For example, Hu et al. proposed a sand cat algorithm incorporating learning behavior that employs cubic B-spline interpolation to generate smooth paths for drone path planning, thereby devising a safe and feasible route while minimizing cost considerations [13]. Addressing the challenge of drone path planning in complex mountainous terrain, Lv et al. proposed a more practical path planning model by comprehensively considering constraints such as natural terrain and collision threats from obstacles, minimum path length, and flight altitude [14]. Building upon the concept of symmetry, they optimized the population initialization method and introduced an improved Beetle Optimization (IDBO) algorithm. This approach effectively reduces UAV energy consumption costs and collision risks while enhancing path planning efficiency and accuracy. Zhang et al. proposed an improved artificial fish school algorithm (IAFSA) to address the issues of uneven population distribution and susceptibility to local optima in traditional artificial fish school algorithms [15], specifically for solving UAV three-dimensional path planning problems. Experimental results demonstrate that IAFSA exhibits high global convergence capability and speed.

However, in line with the No Free Lunch (NFL) theorem, no individual algorithm is universally suitable for solving every problem. Therefore, a great many researchers adjust existing optimization algorithms to fit their particular problem domains, according to the challenges they need to resolve. For example, Maiti, Binanda et al. addressed the limitations of premature convergence and underdevelopment in the traditional Crayfish Optimization Algorithm (COA) [16]. They proposed a novel hybrid optimization algorithm by combining COA with a Differential Evolution (DE) strategy. Extensive experiments were conducted using 34 benchmark functions from CEC 2014 and CEC 2017, along with 6 engineering design problems. HCOADE achieved outstanding results. For the optimization of ship formations and routes, Xu et al. proposed a method combining an improved particle swarm optimization algorithm with a route generation approach based on an enhanced Douglas–Peucker algorithm [17]. The particle swarm algorithm incorporates dynamic adaptive parameter adjustment and genetic algorithm crossover mutation strategies, while the Douglas–Peucker algorithm integrates a density-based noise application spatial clustering algorithm to enhance model performance. Experimental results demonstrate that the improved algorithm outperforms traditional methods in terms of accuracy and stability for fleet target allocation and route planning. Particularly under complex sea conditions, it significantly reduces computational time and errors. In the point cloud registration problem, Fu et al. proposed a modified version of LSHADE-SPACMA (mLSHADE-SPACMA) for numerical optimization and point cloud registration [18]. Experimental results on 25-point cloud registration cases from the Fast Global Registration dataset demonstrate that the modified algorithm achieves competitive results.

Xiao et al. formulated a multi-objective constrained optimization problem considering specific constraints on drone motion, attitude, and altitude, real-world threats such as radar and no-fly zones, and collisions between drones [19]. They developed an Improved Nutcracker Optimization Algorithm to solve this problem. Experimental results demonstrate that the improved Nutcracker Optimization Algorithm outperforms other algorithms in both convergence speed and convergence accuracy, making it an effective method for multi-drone path planning. Liu et al. formulated a multi-drone multi-objective optimization problem for collaborative delivery in urban logistics scenarios [20]. They proposed a non-dominated sorting black-winged kite algorithm to solve this problem, achieving significant improvements in energy efficiency, safety, and computational efficiency. Tang et al. proposed an interval-based multi-objective secretary bird optimization algorithm for three-dimensional modeling of marine environments [21], with hazard levels and navigation time as optimization objectives. Simulation results demonstrate that the proposed algorithm exhibits superior robustness and search capabilities compared to similar algorithms.

The Pelican Optimization Algorithm (POA) is a novel intelligent optimization algorithm inspired by the predatory behavior of pelicans in nature [22]. Proposed in recent years, it has been widely applied to various complex optimization problems, demonstrating significant advantages in fields such as photovoltaic model parameter identification [23], engineering design [24], and robot path planning [25]. Therefore, to address the 3D UAV path planning problem addressed in this paper, based on the above research, this paper proposes a multi-strategy improved POA (MIPOA). The specific contributions are as follows.

1. To enhance the quality of the initial population, a hybrid low-discrepancy and heuristic initialization method is proposed. This method ensures uniform coverage of the search space through low-difference sequences, while heuristics enhance the quality of some individuals and prevent futile searches.
2. We proposed a subgroup mean-guided update strategy to accelerate the algorithm’s convergence toward the global optimum.
3. We propose a random reinitialization boundary control to enhance the algorithm’s exploration capability, enabling it to effectively avoid local optima.
4. The proposed algorithm was evaluated on 30 benchmark functions from the CEC2017 test suite and compared against 11 state-of-the-art metaheuristic algorithms to demonstrate its competitiveness. Furthermore, a comprehensive statistical analysis was conducted to rigorously validate the superior performance of the MIPOA.
5. The MIPOA is applied to the 3D UAV path planning problem under four Scenario s in 3D environment and compared with other comparative algorithms.

The remainder of this paper is structured as follows: Section 2 provides a concise overview of the original POA; Section 3 presents the three enhancement strategies proposed in this study; Section 4 describes the numerical optimization experiments and offers an in-depth analysis of the obtained results; Section 5 applies the proposed MIPOA to the 3D UAV path planning problem and thoroughly discusses its strengths and limitations; finally, Section 6 concludes the paper with a summary of the main findings.

## 2. Pelican Optimization Algorithm (POA)

The Pelican Optimization Algorithm (POA) is a meta-heuristic optimization algorithm inspired by the foraging behavior of pelicans. It has demonstrated excellent performance in solving problems such as Pressure Vessel Design. This paper aims to improve POA based on its existing shortcomings for the UAV path planning problem. Therefore, in this section, we provide a brief introduction to POA, with specific details as follows:

### 2.1. Population Initialization

POA is a population-based algorithm, so population initialization is required before the algorithm begins iteration to obtain the initial solution. In POA, pelicans represent members of the population, with each member representing a candidate solution to the problem. The population matrix of pelicans is denoted by X, specifically expressed as expressed by Equation (1):

(1) $$X={\left[\begin{array}{c} X_{1}\\ \vdots \\ X_{i}\\ \vdots \\ X_{N} \end{array}\right]}_{N\times m}={\left[\begin{array}{ccccc} x_{1,1} & \ldots & x_{1,j} & \ldots & x_{1,m}\\ \vdots & \ddots & \vdots & \ddots & \vdots \\ x_{i,1} & \ldots & x_{i,j} & \ldots & x_{i,m}\\ \vdots & \ddots & \vdots & \ddots & \vdots \\ x_{N,1} & \ldots & x_{N,j} & \ldots & x_{N,m} \end{array}\right]}_{N\times m},$$

where $X_{i}$ denotes the i-th pelican, N denotes the population size, and m denotes the problem dimension. $x_{i,j}$ represents the initial value for each pelican, which is randomly initialized based on the problem’s upper and lower bounds and calculated using Equation (2):

(2) $$x_{i,j}=lb_{j}+rand\cdot \left(ub_{j}-lb_{j}\right),$$

where $ub_{j}$ and $lb_{j}$ represent the upper and lower bounds of the optimization problem, respectively, while rand denotes a random number between 0 and 1.

### 2.2. Moving Towards Prey (Exploration Phase)

After obtaining initialized individuals from the previous phase, the algorithm proceeds to the exploration phase. During this phase, POA explores by simulating movement toward prey, as mathematically expressed by Equation (3):

(3) $$x_{i,j}^{P_{1}}=\left\{\begin{array}{cc} x_{i,j}+rand\cdot \left(p_{j}-I\cdot x_{i,j}\right), & F_{p}<F_{i};\\ x_{i,j}+rand\cdot \left(x_{i,j}-p_{j}\right), & else, \end{array}\right.$$

where $x_{i,j}^{P_{1}}$ is the new position of the i-th pelican in the j-th dimension after position update. $x_{i,j}$ denotes the raw position of the i-th pelican in the j-th dimension. I is a random number whose value is either 1 or 2. p is a randomly selected individual from all pelican populations, representing the prey the pelican needs to find. $p_{j}$ is the prey’s position in the j-th dimension. $F_{p}$ represents the fitness value of the prey, while $F_{i}$ represents the fitness value of $x_{i,j}$. Moreover, more importantly, for pelican individuals in the original population, when the objective function value of a new individual improves, the position of the new individual will be accepted; otherwise, it will not be accepted. This can be specifically expressed by Equation (4):

(4) $$X_{i}=\left\{\begin{array}{cc} X_{i}^{P_{1}}, & F_{i}^{P_{1}}<F_{i};\\ X_{i}, & else, \end{array}\right.$$

where $F_{i}^{P_{1}}$ indicates the fitness value for the new position $x_{i,j}^{P_{1}}$.

### 2.3. Winging on the Water Surface (Exploitation Phase)

Following the exploration phase, the algorithm enters the development phase. At this stage, the POA simulates the behavior of a pelican reaching the water surface, modeling the position update formula as Equation (5):

(5) $$x_{i,j}^{P_{2}}=x_{i,j}+R\cdot \left(1-\frac{t}{T}\right)\cdot \left(2\cdot rand-1\right)\cdot x_{i,j},$$

where $x_{i,j}^{P_{2}}$ denotes the new position of the j-th dimension of the i-th pelican after the Exploitation phase. R is a constant with a value of 0.2, t is the current iteration count, T is the maximum iteration count, $R\cdot \left(1-\frac{t}{T}\right)$ denotes the domain radius of the population, enabling local searches near each member to converge toward better solution. Similarly, at this stage, effective updates are still employed to determine whether to update the positions of pelican individuals in the original population, which can be expressed by Equation (6):

(6) $$X_{i}=\left\{\begin{array}{cc} X_{i}^{P_{2}}, & F_{i}^{P_{2}}<F_{i};\\ X_{i}, & else, \end{array}\right.$$

where $F_{i}^{P_{2}}$ denotes the fitness value for the new position $x_{i,j}^{P_{2}}$. The pseudocode of the POA is outlined in Algorithm 1.
**Algorithm 1:** the pseudo-code of the POA1: ***Begin*** 2: **Initialize:** the relevant parameters and algorithm initialization. 3: While t <T do 4:  **Generate the position of the prey at random.** 5:  **For**
i=1:N 6:   **For**
j=1:M 7:    ***Moving towards prey (exploration phase):*** 8:    Update the population by Equation (3) 9:   **End For** 10:    Update the i−th member in the population by Equation (4) 11:   **End For** 12:   **For**
i=1:N 13:    **For**
j=1:M 14:    ***Winging on the water surface (exploitation phase):*** 15:    Update the population by Equation (5) 16:    **End For** 17:    Update the i−th member in the population by Equation (6) 18:   **End For** 19:   t=t+1 20:   ***Update*** best solution 21: ***End while*** 22: ***return*** best solution 23: ***end***

## 3. Proposed MIPOA

Although POA has demonstrated encouraging results in solving mechanical engineering problems, the NFL theorem asserts that no single algorithm can outperform others across all problems. This theory reveals that an algorithm’s effectiveness depends on its alignment with the specific problem structure. It motivates us to refine algorithms by introducing problem-specific mechanisms tailored to the characteristics of particular problems, thereby enhancing their performance for specific tasks. This principle also serves as one of the theoretical starting points for the continuous evolution and innovation of heuristic algorithms. Therefore, this paper proposes three strategies to address the limitations of POA and enhance its applicability for solving unmanned aerial vehicle (UAV) path planning problems. The specific implementations are as follows:

### 3.1. A Hybrid Low-Discrepancy and Heuristic Initialization Method

In POA, the use of random initialization during the population initialization phase often leads to inconsistent quality of initial solutions, failing to effectively explore promising search spaces. Therefore, we propose a Hybrid Low-Discrepancy and Heuristic Initialization Method that generates initial solutions with more uniform coverage of the search space compared to random initialization, thereby enhancing the algorithm’s global exploration quality. Furthermore, heuristic solutions can immediately introduce high-quality feasible solutions, elevating the average fitness of the initial population and accelerating algorithm convergence. By combining both approaches, we achieve a balance between exploration and exploitation, reducing ineffective early-stage searches while preserving the ability to discover global solutions. Its mathematical model can be expressed by Equation (7):

(7) $$X=X_{\mathrm{LDS}}\cup X_{H}\cup X_{R},$$

where $X_{\mathrm{LDS}}$ denotes the initial solution obtained from the low-variance sampling component, $X_{H}$ denotes the initial solution generated by the heuristic component, and $X_{R}$ denotes the initial solution generated by the random component. The calculation method can be performed using Equations (8)–(10):

(8) $$x_{j}^{\mathrm{LDS}}=lb_{j}+u_{j}⊙\left(ub_{j}-lb_{j}\right),$$

(9) $$x_{k}^{H}=H\left(\theta_{k}\right),$$

(10) $$x_{m,j}^{R}∼U\left(lb_{j},ub_{j}\right).$$

Additionally, the number of individuals in populations $X_{\mathrm{LDS}}$, $X_{H}$, and $X_{R}$ can be expressed as $N_{\mathrm{LDS}}$, $N_{H}$, and $N_{R}$, $N_{\mathrm{LDS}}+N_{H}+N_{R}=N$. $u_{j}$ is a random point obtained through Latin hypercube sampling, with initial individuals derived via upper and lower bound mapping of the problem. $H\left(\theta_{k}\right)$ is a heuristic rule function that can be calculated using Equation (11). This mapping ensures that the population contains boundary points and center points:

(11) $$H\left(\theta \right)=lb_{j}+\theta \left(ub_{j}-lb_{j}\right),\theta \in \left\{0,0.5,1\right\},$$

where $U\left(lb_{j},ub_{j}\right)$ indicates uniform distribution, meaning random sampling over the interval $[lb_{j}$, $ub_{j}]$.

### 3.2. A Subgroup Mean-Guided Update Strategy

In the two phases of POA, position updates depend on both the individual’s current location and the prey’s location, with the latter achieved through randomization. While simultaneously utilizing both current location and prey location enables the algorithm to explore effectively, it may fail to fully leverage information from high-quality solutions, resulting in slower convergence. To address this limitation of POA, we propose a subpopulation mean-guided update strategy. In this approach, we partition a portion of the original population into two subpopulations. By effectively leveraging information provided by individuals within each subpopulation, we accelerate algorithmic convergence. The specific position update formula can be modeled as Equation (12):

(12) $$x_{i}^{new}=\left\{\begin{array}{cc} x_{i}+rand\cdot \left(Xp_{mean}-x_{r2}\right), & r_{k}<cs;\\ x_{i}+rand\cdot \left(Xq_{mean}-x_{r2}\right), & else, \end{array}\right.$$

where $x_{i}^{new}$ denotes a new individual generated via the subgroup mean-guided update strategy, while $r_{k}$ represents a random number between 0 and 1 used to determine which subpopulation to employ for position updates. cs is a constant set to 0.5 to balance the utilization frequency of both subpopulations, achieving equilibrium between exploration and exploitation in the MIPOA. r2 is a random integer used to select random individuals within the population, ensuring the algorithm’s exploratory nature prevents it from becoming overly prone to local optima. rand represents a random number between 0 and 1. $x_{i}$ indicates the original position of individual i. $Xp_{mean}$ and $Xq_{mean}$ represent the two populations we have classified, whose mathematical models can be expressed as Equations (13) and (14):

(13) $$Xp_{mean}=\left\{x_{1},x_{2},\ldots ,x_{p}\right\},\;\;x_{p}\in X,$$

(14) $$Xq_{mean}=\left\{x_{1},x_{2},\ldots ,x_{q}\right\},\;\;x_{q}\in X,$$

where p and q represent the number of individuals in subpopulations $Xp_{mean}$ and $Xp_{mean}$. Since the 3D UAV path planning problem addressed in this paper constitutes a complex optimization challenge, the algorithm must possess strong capabilities to escape local optima. Therefore, in our subpopulation mean-guided update strategy, to enhance the algorithm’s randomness, we employ random integers to determine the size of the two subpopulations. This approach increases the algorithm’s randomness and strengthens its ability to explore diverse solutions. The value of p is a random integer between 2 and 10, while the value of q is a random integer between 10 and N. These values are updated during each iteration to control the sizes of the two subpopulations. Individuals in both subpopulations are randomly selected, non-replicated individuals from the original population.

### 3.3. A Random Re-Initialization Boundary Control

**Figure 1:**
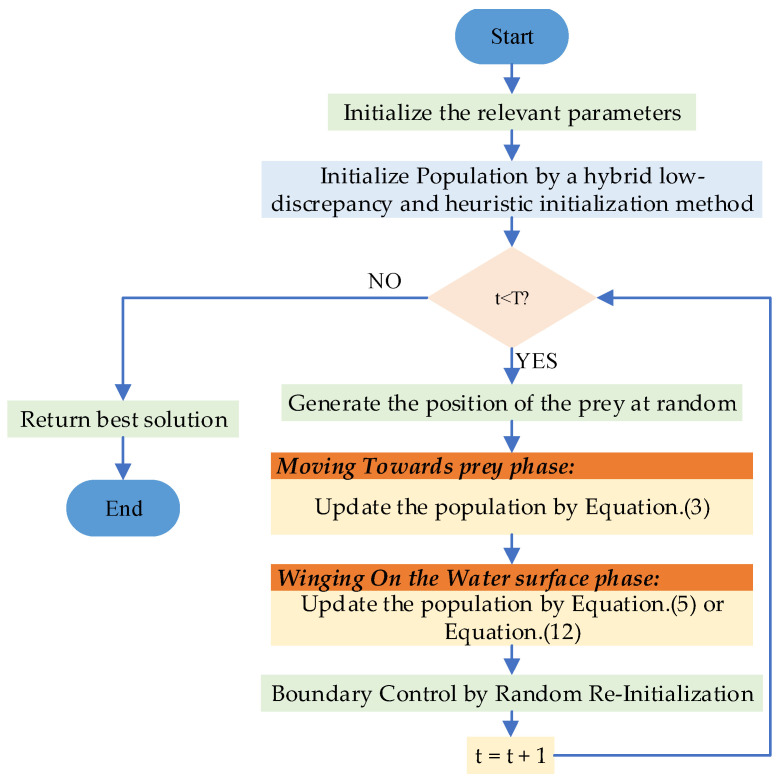
Flowchart of MIPOA.

For the POA, although its position updates are based on information such as subpopulations and the current optimal solution, making position overruns less likely, the nature of the random factor may occasionally cause updates to exceed the predefined search space, leading to failure of the current algorithm iteration. Therefore, this section proposes a random boundary reset control mechanism to reprocess out-of-bounds individuals. When an individual’s position exceeds the boundary, the system does not directly skip the update operation. Instead, it randomly regenerates a feasible solution within the search space. This approach ensures individuals remain within the valid region while injecting new randomness into the population, thereby enhancing diversity and reducing the probability of premature convergence. Its mathematical model is expressed by Equation (15). Figure 1 presents the flowchart of the MIDOA algorithm. The pseudocode of the MIPOA is outlined in Algorithm 2:

(15) $$x_{i,j}=\left\{\begin{array}{c} \mathrm{rand}\left(lb_{j},ub_{j}\right),\;\;\mathrm{if}\;x_{i,j}<lb_{j}\;\mathrm{or}\;x_{i,j}>ub_{j}\\ x_{i,j},\;\;\mathrm{otherwise} \end{array}\right..$$

**Algorithm 2:** The pseudo-code of the MIPOA1: ***Begin*** 2: Initialize population by a hybrid low-discrepancy and heuristic initialization method. 3: While t<T do 4:  **Generate the position of the prey at random.** 5:  **For**
i=1:N 6:   **For**
j=1:M 7:    ***Moving towards prey (exploration phase):*** 8:    Update the population by Equation (3) 9:   **End For** 10:    Update the i−th member in the population by Equation (4) 11:   **End For** 12:   **For**
i=1:N 13:    **For**
j=1:M 14:    ***Winging on the water surface (exploitation phase):*** 15:    Update the population by Equation (12) 16:    **End For** 17:    Update the i−th member in the population by Equation (6) 18:   **End For** 19:   Boundary Control by Random Re-Initialization 20:   t=t+1 21:   ***Update*** best solution 22: ***End while*** 23: ***return*** best solution 24: ***end***

### 3.4. Time Complexity Analysis

Time complexity analysis plays a crucial role in the study of intelligent optimization algorithms. By analyzing the time complexity, researchers can quantitatively evaluate the computational cost of the algorithm when solving problems of varying scales, thereby reflecting its efficiency and scalability. Therefore, in this subsection, we analyze the time complexity of MIPOA. The time complexity of MIPOA primarily consists of population initialization, position update, and boundary control. For population initialization, the position of each individual must be initialized, with a time complexity of $O\left(N\times M\right)$, for position updates, each individual’s position must be updated within the main loop. The time complexity of position updates is $O\left(T\times N\times M\right)$, after a position update, boundary control is required, so the time complexity of boundary control is also $O\left(T\times N\times M\right)$, In summary, the time complexity of MIPOA is $O\left(T\times N\times M\right)$, identical to that of POA. This demonstrates that our improvements did not increase the algorithm’s complexity by an order of magnitude.

## 4. Global Optimization Experimental and Detailed Analyses

In this section, to evaluate the performance of MIPOA, we conducted comprehensive testing using 30 test functions from the CEC2017 test set. First, we briefly introduce the test functions, comparison algorithms, and parameter settings. We then perform a thorough analysis of population diversity and convergence performance. Finally, we conduct a statistical analysis to assess the significance of MIPOA compared to other comparison algorithms. Detailed specifics are as follows:

### 4.1. Benchmark Test Functions

The CEC2017 benchmark dataset, proposed by IEEE at the 2017 Conference on Evolutionary Computation, is widely used to evaluate the performance of single-objective, continuous-domain, boundary-constrained optimization algorithms [26]. This dataset comprises 30 complex test functions subjected to offset and rotation processing. The search space is typically defined on the ${\left[-100,\;100\right]}^{D}$, with common dimensions being 10, 30, 50, and 100. Based on their characteristics, the functions are categorized into unimodal, multimodal, hybrid, and composite functions. These categories, respectively, evaluate an algorithm’s convergence speed, global exploration capability, robustness, and overall adaptability. Monotone functions emphasize local development capability, multi-peak functions contain numerous local extrema, hybrid functions combine different functional structures across subspaces, and composite functions construct complex multi-level topographies through weighted combinations. Consequently, this test set presents a highly challenging environment. In summary, the CEC2017 test set is selected as the benchmark for evaluating MIPOA performance. The details of 30 test functions in the CEC2017 are listed in Table A1.

### 4.2. Competitor Algorithms and Parameters Setting

In this section, the performance of the proposed MIPOA is assessed by comparing it with eleven state-of-the-art metaheuristic algorithms to highlight its optimization capability. The algorithms used for comparison include Particle Swarm Optimization (PSO), Snake Optimizer (SO), Chameleon Swarm Algorithm (CSA), Dwarf Mongoose Optimization Algorithm (DMO), Artificial Gorilla Troops Optimizer (GTO), Rime optimization algorithm (RIME), Information acquisition optimizer (IAO), Red-tailed hawk algorithm (RTH), Black-winged Kite Algorithm (BKA), Genghis Khan Shark Optimizer (GKSO), and Pelican Optimization Algorithm (POA). Table 1 summarizes the parameter settings of these algorithms for easier reading.

### 4.3. Analysis of the Population Diversity

Population diversity analysis evaluates the diversity level of a population by measuring the distribution differences in individuals within the solution space, thereby reflecting an algorithm’s ability to maintain exploration capacity and avoid premature convergence during the search process [37]. For optimization algorithms, population diversity analysis holds significant importance: on one hand, it reveals the search characteristics of the algorithm at different iteration stages, such as the balance between global exploration and local exploitation; on the other hand, it helps determine whether the algorithm has fallen into a local optimum and assesses its convergence stability. Therefore, conducting population diversity analysis on optimization algorithms not only deepens our understanding of the algorithm’s search mechanism but also provides a theoretical basis for algorithm improvement and parameter tuning. Therefore, in this subsection, we assess the population diversity of the MIPOA, which is calculated using Equation (16):

(16) $$I_{C}\left(t\right)=\sqrt{\sum_{i=1}^{N}\sum_{d=1}^{m}\;{\left(x_{id}\left(t\right)-c_{d}\left(t\right)\right)}^{2}},$$

where $I_{C}\left(t\right)$ denotes the population diversity, and $x_{id}\left(t\right)$ denotes the value of the i individual in the d dimension at the t iteration. $c_{d}\left(t\right)$ reflects the degree of dispersion of the entire population relative to the center of mass at the t iteration. $c_{d}\left(t\right)$ is calculated using Equation (17):

(17) $$c_{d}\left(t\right)=\frac{1}{D}\sum_{i=1}^{N}\;x_{id}\left(t\right).$$

**Figure 2:**
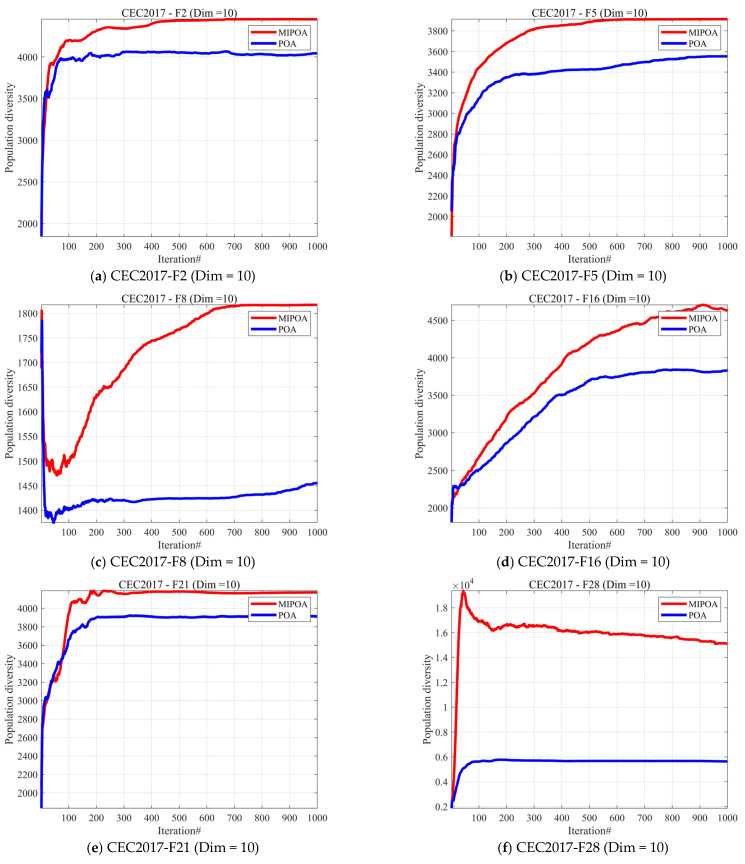

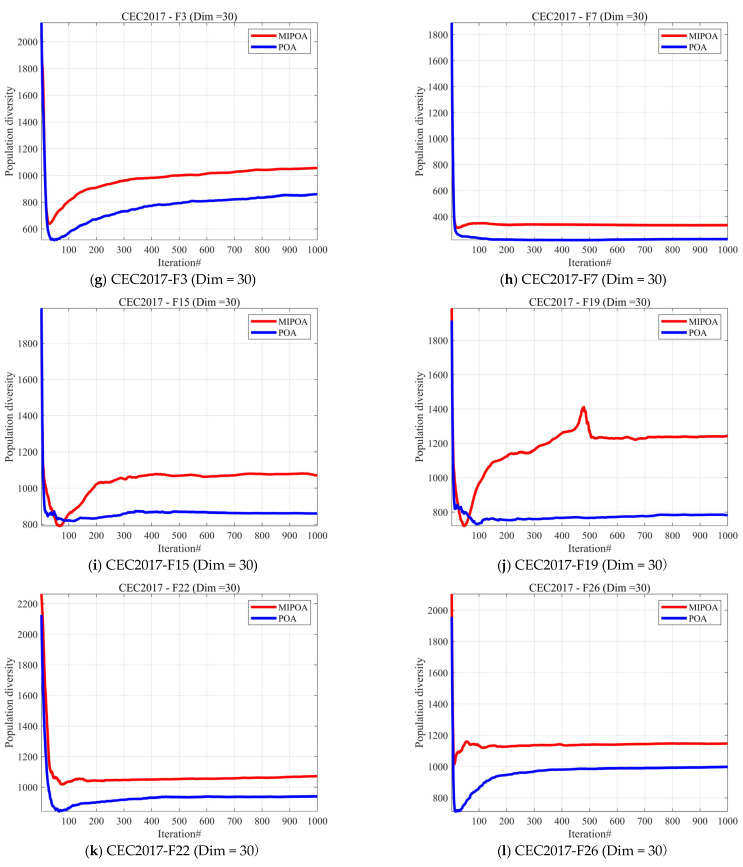

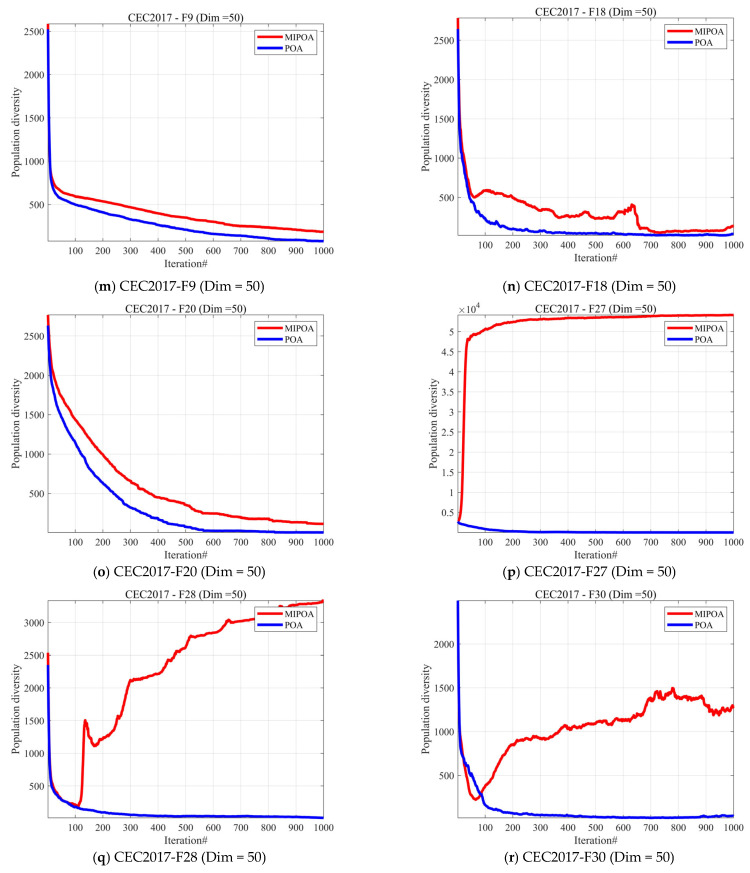
The analysis of the population diversity of MIPOA and POA.

Figure 2 presents the experimental results of the MIPOA and POA across three dimensions on the CEC2017 test set. Overall, MIPOA maintains significantly higher diversity levels than POA across all evaluation metrics. Notably, in the 10-dimensional setting, the gap between MIPOA and POA is particularly pronounced in F16 and F28, demonstrating its superior global exploration capability and ability to escape local optima. In contrast, POA’s diversity rises rapidly in the early stages before stabilizing at a relatively low overall level, posing a risk of premature convergence. In the 30-dimensional setting, MIPOA demonstrated overall higher population diversity compared to POA, with a more pronounced advantage on complex and multi-modal functions (e.g., F19). This indicates its superior global exploration capability and effectiveness in avoiding premature convergence. However, the gap was smaller on some simpler functions (e.g., F7), suggesting that the enhancement of population diversity under high-dimensional conditions is more prominent in complex scenarios.

In the 50-dimensional problem, MIPOA maintains higher population diversity compared to POA, particularly excelling on complex functions such as F18, F20, and F27. The results for F27 demonstrate MIPOA’s strong global exploration advantage in high-dimensional complex environments, whereas POA rapidly converges while maintaining low diversity, making it prone to premature convergence. Overall, MIPOA demonstrates greater robustness and adaptability under high-dimensional conditions, effectively mitigating the premature convergence issues inherent in traditional POA. Table 2 presents the population diversity indices of the two algorithms on the 30-dimensional CEC2017 test set. The experimental results demonstrate that MIPOA achieves superior population diversity indices compared to POA on most functions.

### 4.4. Parameter Sensitivity Analysis

**Figure 3:**
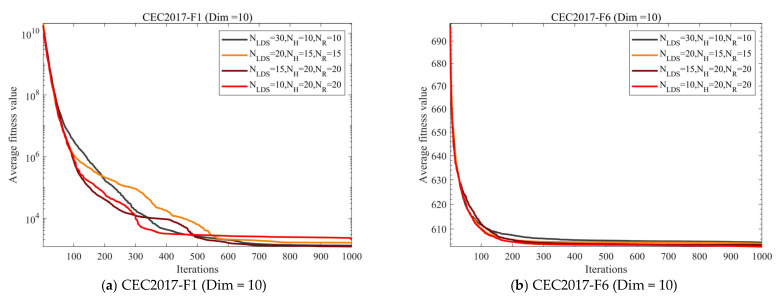

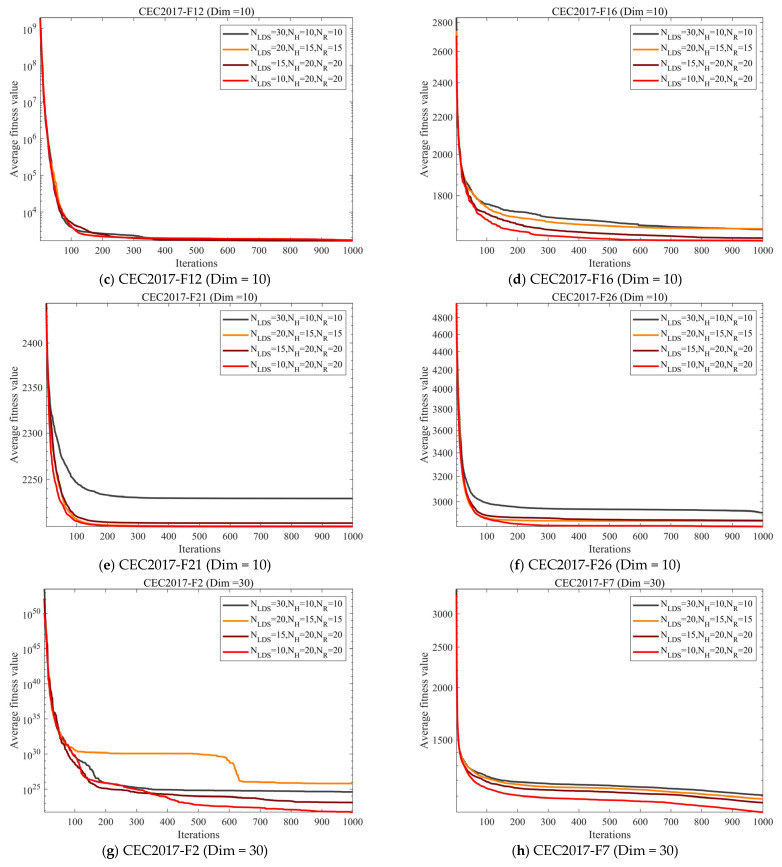

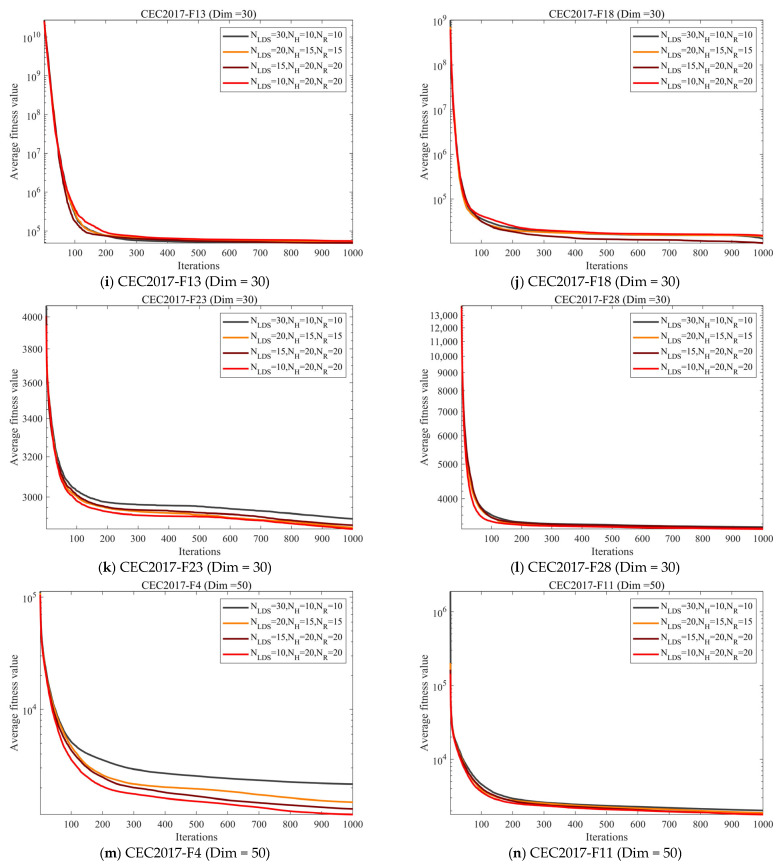

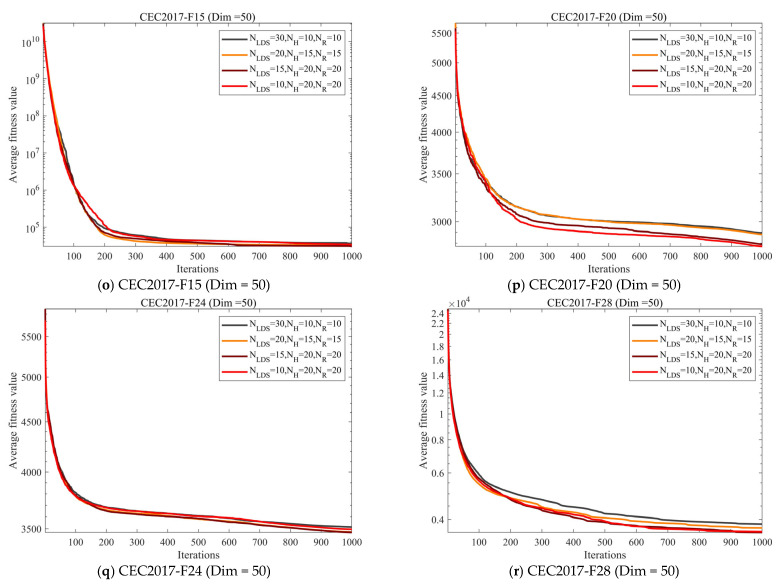
The analysis of the Parameter Sensitivity.

Parameter sensitivity analysis holds significant importance for studying the performance of heuristic algorithms. Heuristic algorithms typically rely on multiple control parameters to balance global search capabilities with local exploration abilities. Different parameter settings can significantly impact the algorithm’s convergence speed, stability, and solution accuracy. By conducting sensitivity analysis on key parameters, we can systematically evaluate their impact on algorithmic performance, thereby determining reasonable parameter ranges to enhance robustness and adaptability. Consequently, this subsection performs parameter sensitivity analysis on MIPOA, with experimental results presented in Figure 3.

For the number of initialized individuals, we conducted parameter sensitivity analysis across four scenarios. Experimental results demonstrate that our algorithm consistently achieves favorable outcomes across all four cases, converging to optimal solutions within a limited number of iterations.

### 4.5. Compare Using CEC 2017 Test Functions

**Figure 4:**
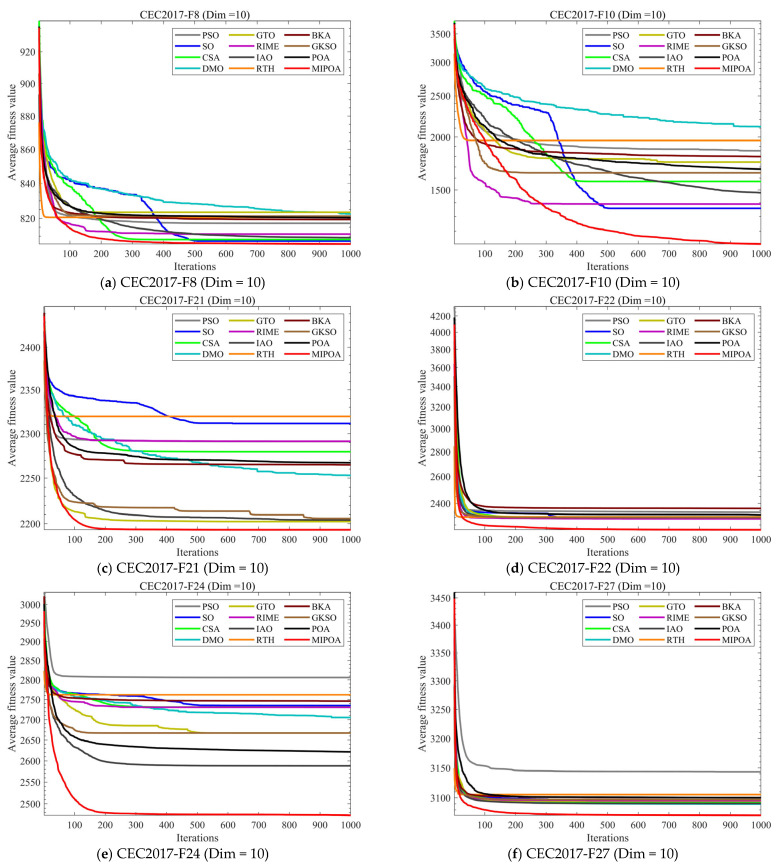

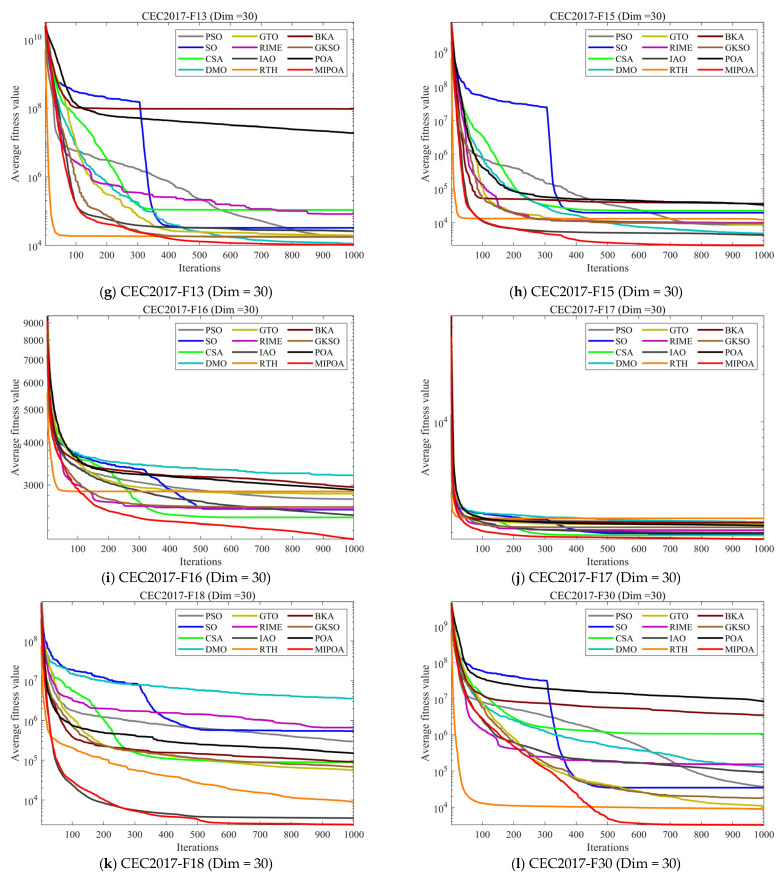

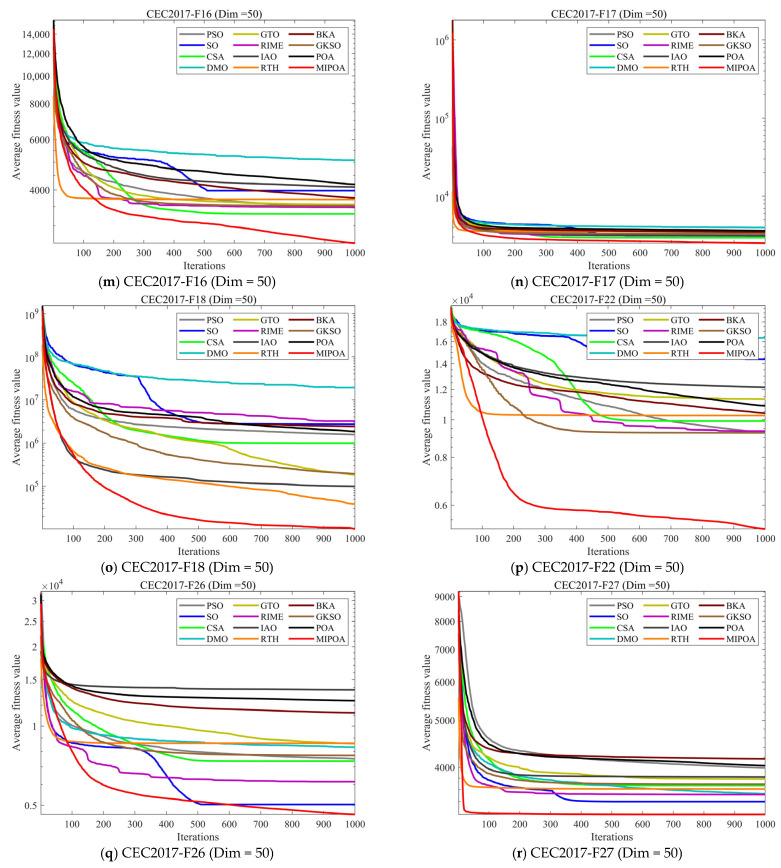
Comparison of convergence speed of different algorithms on CEC2017 test set.

To evaluate MIPOA’s performance, we assessed it using the CEC2017 test set. Comparisons were made against 11 other state-of-the-art algorithms, with experimental analysis conducted using the mean and standard deviation from 30 independent runs. Furthermore, to compare the convergence rates of each algorithm, Figure 3 presents the experimental results of MIPOA and the comparison algorithms across three dimensions on the CEC2017 test set. To further analyze the algorithm’s performance, Figure 4 displays box plots from 30 independent runs, providing a comprehensive analysis of the results from each run. Table A2, Table A3, and Table A4, respectively, present the performance metrics of the 12 algorithms across three dimensions on the CEC2017 test set. Here, “mean” denotes the average of 30 independent runs, while “std” represents the standard deviation of the algorithm’s 30 independent runs. The data in the table indicates that MIPOA achieved the best results among the 12 algorithms, with the most significant improvement observed when compared to POA.

As can be seen from Figure 4, the MIPOA demonstrates outstanding convergence advantages: First, it exhibits a rapid convergence rate during the early stages of iteration, with the average fitness value decaying quickly. Second, it converges stably to a low fitness level in the later stages of iteration, reflecting strong optimization accuracy and convergence stability. In contrast, algorithms such as PSO, SO, and CSA exhibit slower convergence speeds on certain functions and converge to relatively high final fitness values, resulting in comparatively inferior convergence performance. It is worth noting that for simple functions, most existing algorithms exhibit excellent performance, finding the optimal solution in a relatively small number of iterations. Therefore, MIPOA’s performance improvement on simple functions is limited. However, for complex functions, existing algorithms often struggle to find the global optimum. This is where MIPOA’s advantage becomes apparent, as it effectively escapes local optima to achieve the global optimum. More importantly, the three-dimensional UAV path planning problem addressed in this paper is a complex problem, making the performance improvement of MIPOA on complex functions even more significant.

**Figure 5:**
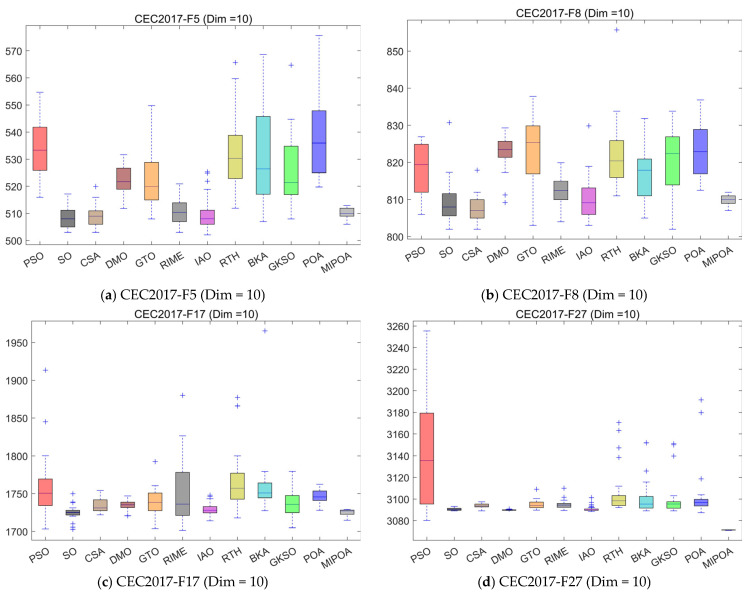

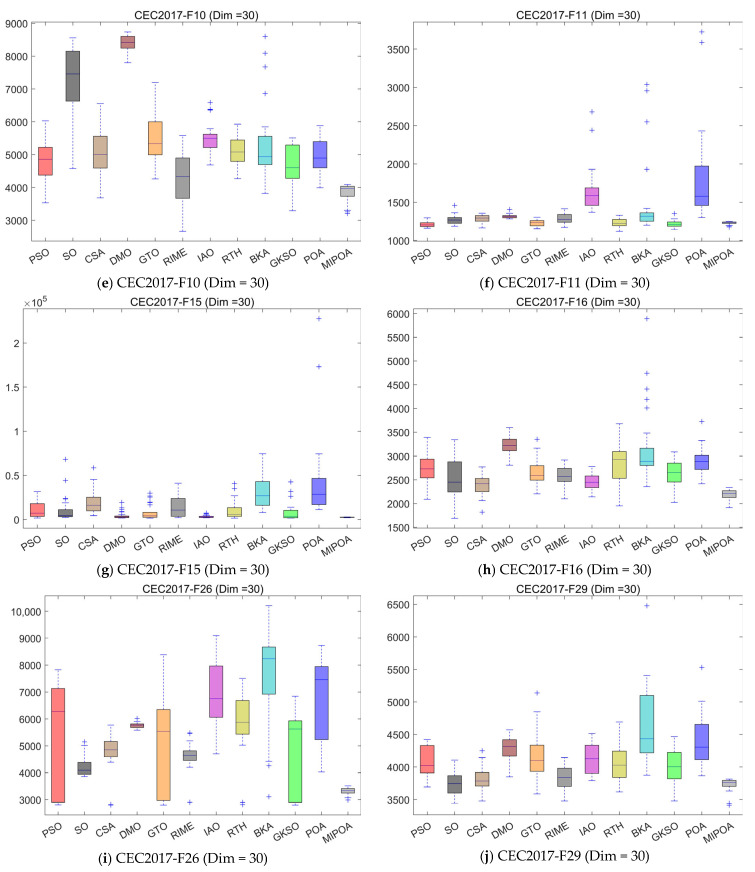

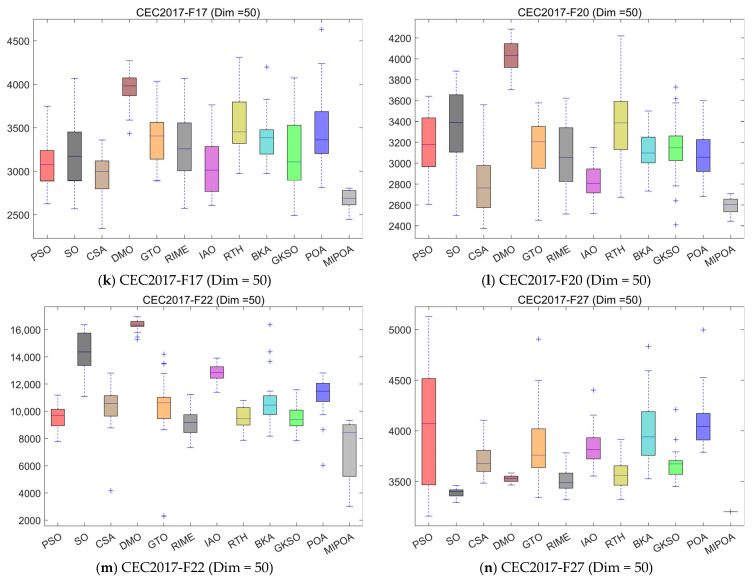
Boxplot comparison of different algorithms on the CEC2017 test suite.

Figure 5 demonstrates significant differences in performance stability and optimization accuracy among various intelligent optimization algorithms. The MIPOA exhibits outstanding performance across multiple experimental sets: First, its box plot median is significantly lower than other algorithms, indicating a clear advantage in average optimization accuracy. Second, the box plot’s interquartile range is narrow with minimal outliers, reflecting exceptional performance stability. In contrast, algorithms like PSO, SO, and RTH exhibit higher box plot medians. Some algorithms (e.g., PSO on functions F5 and F10, POA on function F11) display large box plot spans and numerous outliers, indicating suboptimal performance in both optimization accuracy and stability. In summary, MIPOA demonstrates greater competitiveness in the comprehensive performance of optimization accuracy and stability when handling complex optimization problems across different dimensions.

### 4.6. Statistical Analysis

To determine whether significant differences exist between MIPOA and each comparison algorithm, this section conducts statistical analysis on all algorithms. First, the Wilcoxon signed-rank test is applied to evaluate the 12 algorithms and compare their significant differences. Subsequently, the Friedman test of ranks is employed to assess the ranking performance of each algorithm. Specific details are as follows:

#### 4.6.1. Wilcoxon Rank Sum Test

In this section, we conducted the Wilcoxon rank sum test on 12 algorithms [38]. The Wilcoxon Rank Sum Test (Mann–Whitney U Test) is a commonly used nonparametric statistical test primarily employed to compare whether the overall distributions of results from two algorithms exhibit significant differences. In this experiment, we set the significance level to 0.05. If p<0.05, we reject the null hypothesis, indicating a significant difference; otherwise, we accept the null hypothesis, suggesting no significant difference. Table A5, Table A6 and Table A7 present the experimental results of 11 comparison algorithms versus MIPOA across three dimensions on the CEC2017 test set.

The experimental results indicate that, at a significance level of α = 0.05, MIPOA exhibits highly statistically significant differences (*p*-values far below 0.05) from PSO, CSA, DMO, GTO, RIME, BKA, GKSO, and RTH in the vast majority of test items (F1–F30). It showed no significant difference with SO only in test item F5, while significant differences were observed in all other items. With IAO, no significant differences were found only in 6 test items (F7, F8, F12, F20, F23, F29), while significant differences were observed in all other items. In summary, MIPOA’s performance is statistically significantly superior to (or distinct from) the vast majority of comparison algorithms, with only a few test items showing no significant difference compared to SO and IAO. Its algorithmic effectiveness is strongly supported by quantitative statistical evidence.

#### 4.6.2. Friedman Mean Rank Test

The Friedman Mean Rank Test is a nonparametric statistical test used to analyze whether there are significant differences among the experimental results of multiple algorithms. It is particularly commonly used to compare the effectiveness differences in three or more treatment methods on the same test subjects. Therefore, in this section, we employ the Friedman Mean Rank Test to evaluate the performance of 12 algorithms and obtain their rankings. The experimental results are shown in Table 3. Among these, M.R denotes the average ranking of the 12 algorithms across 30 test functions, while T.R represents the final ranking of the 12 algorithms across 30 test functions.

Based on experimental results across different dimensionality levels (10-D, 30-D, 50-D) of the CEC2017 test set, MIPOA demonstrates significant and comprehensive performance advantages. On the two key metrics of average rank and final rank, MIPOA achieved an average rank of 2.27 and a final rank of 1 in the 10-dimensional test, significantly outperforming comparison algorithms like PSO and SO, demonstrating outstanding performance in low-dimensional scenarios. In the 30-dimensional test, it maintained optimal performance with an average rank of 3.30 and a final rank of 1, showing a clear advantage over algorithms like POA and BKA in medium-dimensional scenarios. Even in the more demanding 50-dimensional test requiring higher algorithmic complexity and optimization capability, MIPOA achieved an average rank of 4.33 and a final rank of 1, substantially outperforming algorithms like POA and IAO with robust generalization ability. Furthermore, MIPOA maintained absolute leadership in both average and final ranks across all dimensionality levels. Compared to the unmodified POA, MIPOA significantly reduces the average rank from POA’s 8.97 (10 dimensions), 10.23 (30 dimensions), and 10.40 (50 dimensions) to 2.27, 3.30, and 4.33, demonstrating markedly enhanced optimization performance. In summary, MIPOA demonstrated its efficiency and stability across optimization problems of varying complexity on the CEC2017 test set in 10-, 30-, and 50-dimensional scenarios, achieving the smallest average rank and optimal final rank. It exhibits clear performance advantages over other comparison algorithms.

## 5. MIPOA for 3D UAV Path Planning

In the previous section, we evaluated MIPOA using the CEC2017 test set. In this section, to plan drone flight trajectories in a three-dimensional environment, we establish the drone’s flight environment through a 3D map model. Subsequently, we construct a fitness function based on factors such as path length and altitude cost and conduct analysis through simulation experiments. The specific implementation steps are as follows.

### 5.1. Scenarios and Objective Functions

In this section, we model the drone’s flight environment and objective function, with detailed specifications as follows:

#### 5.1.1. Scenario Setting

**Figure 6:**
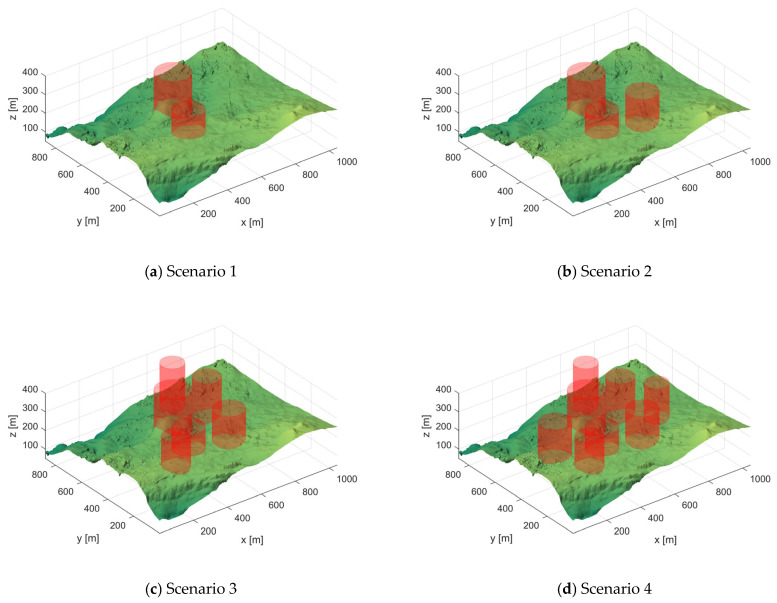
Four different scenario views.

When constructing the experimental environment in this section, we first used a real-world digital elevation model map captured by LiDAR as the base. Then, cylindrical objects were employed to simulate actual obstacles such as trees, resulting in four distinct drone flight scenarios. Figure 6 illustrates these four scenarios, each featuring varying densities of obstacle distribution, in our schematic diagram, the green areas represent the basic mountainous terrain, while the red cylinders simulate real-world obstacles such as trees. This setup comprehensively simulates the diverse environmental threats drones may encounter in real-world conditions.

#### 5.1.2. Optimization Problem Definition

This section first defines the path cost, threat cost, altitude cost, and smoothness cost of UAV flight. Finally, it combines these four objectives to define the optimization function for UAV path planning. Specific details are as follows:

1. Path Length Costs

In UAV flight path planning, path cost serves as the core quantitative metric for evaluating the “quality” of a flight path, directly determining the rationality and practicality of the route design. For small and medium-sized UAVs with limited endurance, flight distance stands as one of the primary optimization objectives. In this subsection, we assume that the flight waypoint of the UAV is $P_{ij}=\left(x_{ij},y_{ij},z_{ij}\right)$. The Euclidean distance between two waypoints is $\vec{P_{ij}P_{i,j+1}}$. Therefore, the flight cost of the UAV is given by Equation (18):

(18) $$F_{1}\left(X_{i}\right)=\sum_{j=1}^{n-1}\;∥\vec{P_{i,j}P_{i,j+1}}∥.$$

2. Threat Costs

During drone flight, static obstacles encountered along the flight path—such as buildings and mountains—along with dynamic threats like other aircraft, all constitute obstacle hazards. Threat Costs serve as a key metric for assessing flight path safety, quantifying the potential consequences of various threats a drone may face during operation. In this subsection, the primary threats during drone flight include obstacles simulating environmental features like trees. Based on this, the threat cost of UAV is calculated by Equation (19):

(19) $$F_{2}\left(X_{i}\right)=\sum_{j=1}^{n-1}\;\sum_{k=1}^{K}\;T_{k}\left(\vec{P_{ij}P_{i,j+1}}\right),$$

where $T_{k}\left(\vec{P_{ij}P_{i,j+1}}\right)$ denotes flight constraint costs, which is calculated by Equation (20):

(20) $$T_{k}\left(p_{ij}p_{i,j+1}\right)=\left\{\begin{array}{cc} 0, & d_{k}>S+D+R_{k}\\ \left(S+D+R_{k}\right)-d_{k}, & D+R_{k}<d_{k}\leq S+D+R_{k}\\ \infty , & d_{k}\leq D+R_{k} \end{array}\right.,$$

where $R_{k}$ denotes the radius of the Kth cylindrical obstacle, D denotes the peripheral collision region, and $d_{k}$ denotes the distance from the center of the obstacle to the path $L_{p_{ij}p_{i+1,j}}$.

3. High Costs

In drone path planning, altitude cost serves as the core metric for evaluating the “altitude rationality” of flight paths. Natural environments and airspace regulations within the flight zone impose “hard constraints” on altitude, with violations incurring “compliance risk costs.” For instance, the optimal altitude range for urban delivery drones is 50–80 m, while inspection drones in plateau regions operate best within 30–50 m. Therefore, the altitude cost of the UAV is calculated by Equation (21):

(21) $$F_{3}\left(X_{i}\right)=\sum_{j=1}^{n}\;H_{ij},$$

where $H_{ij}$ denotes the cost of the height of the $X_{i}$ location, which is calculated by Equation (22):

(22) $$H_{ij}=\left\{\begin{array}{ll} |h_{ij}-\frac{\left(h_{max}+h_{min}\right)}{2}|, & h_{min}\leq h_{ij}\leq h_{max}\\ \infty , & \mathrm{otherwise} \end{array}\right.,$$

where $h_{ij}$ is the altitude at which the UAV is located; $h_{min}$ is the minimum altitude at which flight is allowed; and $h_{max}$ is the maximum altitude at which flight is allowed.

4. Smoothness Costs

In UAV path planning, smoothness cost serves as a key metric for evaluating the “motion continuity and stability” of flight paths. The smoother the path (with gentler turns, climbs, and descents), the simpler the drone’s attitude control becomes, resulting in lower energy consumption and more stable mission execution. Conversely, paths with abrupt fluctuations incur additional control costs, energy consumption costs, and risk costs. Therefore, the smoothing cost for the UAV flight is calculated by Equation (23):

(23) $$F_{4}\left(X_{i}\right)=a_{1}\sum_{j=1}^{n-2}\;\alpha_{ij}+a_{2}\sum_{j=1}^{n-1}\;\left|\beta_{ij}-\beta_{i,j-1}\right|,$$

where $a_{1}$ denotes the UAV horizontal turn angle constraint penalty coefficient, and $a_{2}$ denotes the UAV vertical pitch angle constraint penalty coefficient. $\alpha_{ij}$ denotes the horizontal turn angle constraint, which is computed by Equation (24). $\beta_{ij}$ denotes the vertical pitch angle, which is computed by Equation (25):

(24) $$\alpha_{ij}=\arctan\frac{L_{p_{ij}^{\prime }p_{i,j+1}^{\prime }}\times L_{p_{ij}^{\prime }p_{i,j+2}^{\prime }}}{L_{p_{ij}^{\prime }p_{i,j+1}^{\prime }}\cdot L_{p_{ij}^{\prime }p_{i,j+2}^{\prime }}},$$

(25) $$\beta_{ij}=\arctan\frac{Z_{i,j+1}-Z_{ij}}{∥L_{p_{ij}p_{i,j+1}}∥},$$

where $L_{p_{ij}^{\prime }p_{i,j+1}^{\prime }}$ is their projections on the plane, which is calculated by Equation (26).

(26) $$L_{p_{ij}^{\prime }p_{i,j+1}^{\prime }}=k\times \left(L_{p_{ij}p_{i,j+1}}\times k\right),$$

where k is the unit vector in the positive direction of the axis.

5. Overall Objective Function (OEF)

Considering the path length cost, threat cost, high cost, and smoothness cost, the overall objective function based on multi-cost is calculated by Equation (27):

(27) $$F\left(X_{i}\right)=\sum_{k=1}^{4}{b_{k}*F}_{k}\left(X_{i}\right),$$

where $b_{k}$ is the weight coefficient. Following the work of Xie et al., in UAV path planning, path cost is the most critical factor, as path length directly impacts mission execution speed and energy consumption. Therefore, we set the path cost coefficient to 0.5. However, threat cost is also extremely important for UAVs; if a UAV cannot effectively avoid threats, it cannot safely execute its mission. Thus, we set the threat cost coefficient to 0.3, while height cost and smoothness cost are each set to 0.1.

#### 5.1.3. Analysis of Experimental Results

**Figure 7:**
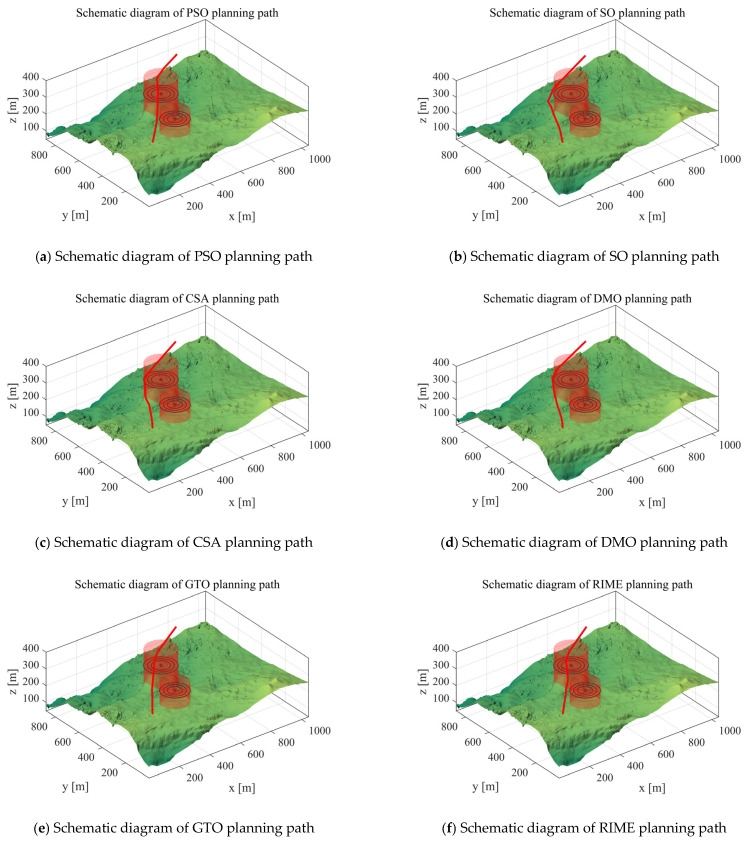

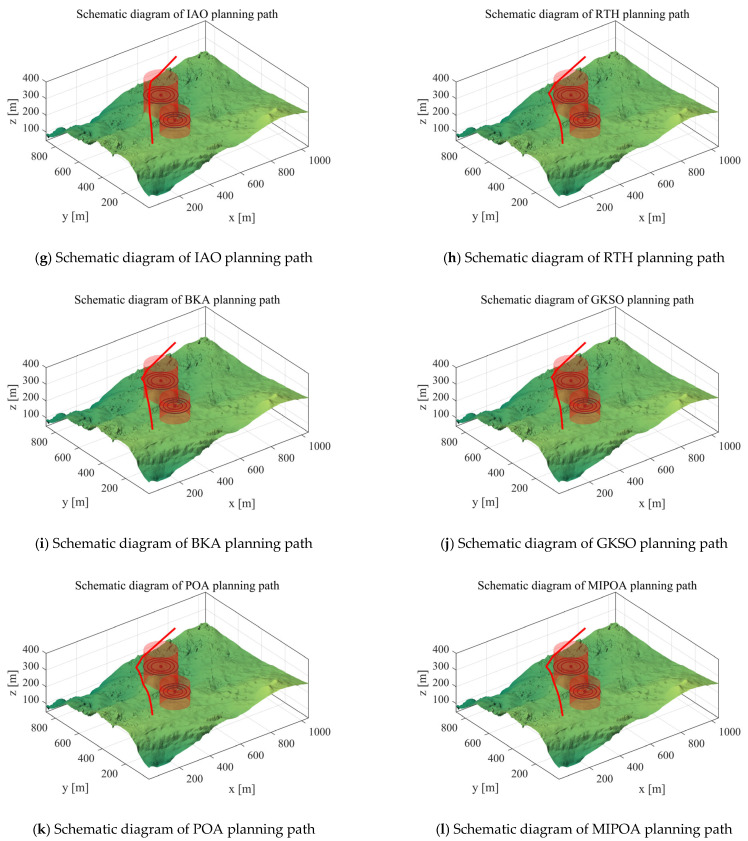
Scenario 1 Schematic diagram of path planning.

To carry out a thorough analysis of MIPOA, we conducted experimental studies in four scenarios, and the specific details are as follows:

*Scenario 1*: We utilized two cylindrical obstacles to mimic real-world hazards and adopted simulation experiments to verify the UAV path planning performance of the MIPOA under two cylindrical threat scenarios. In the experimental configuration, the UAV’s starting position was set to [100, 100, 150], the destination to [800, 800, 150], and the number of waypoints was set as 10. The total path costs are presented in Table 4: “Mean” stands for the average value of 30 independent operation runs, “Max” refers to the highest cost recorded across the 30 algorithm runs, and “Min” represents the optimal cost achieved in the 30 runs. Figure 7 displays the paths planned for the UAV by each algorithm in Scenario 1. The red line indicates the flight path planned for the UAV.

The experimental results demonstrate that MIPOA significantly outperforms the comparison algorithms PSO, SO, CSA, DMO, GTO, RIME, IAO, RTH, BKA, GKSO, and POA in overall performance. Regarding the “mean” metric, MIPOA achieved an average value of 416.01. This figure not only falls significantly below the 455.47 recorded by the classical PSO algorithm but also underperforms compared to POA’s 419.11. Furthermore, its mean value remains lower than that of other algorithms such as SO and GTO. This indicates that over 30 independent runs, MIPOA achieves lower average path costs, demonstrating superior algorithmic stability and overall optimization capability. Regarding the “max” metric, MIPOA’s maximum value is only 416.34—the lowest among all algorithms. In contrast, RTH’s maximum reaches 458.59, while PSO reached 501.23—both significantly higher than MIPOA. Even compared to POA’s 424.53, MIPOA’s path cost in worst-case scenarios remains lower. This fully demonstrates the algorithm’s superior robustness in complex scenarios, where it is less prone to extreme deviations from optimal solutions. Examining the “min (minimum value)” metric, which reflects an algorithm’s potential for optimal performance, MIPOA achieved a minimum of 414.90. While not the absolute lowest among all algorithms (e.g., SO’s minimum was 414.70, slightly below MIPOA), it remains exceptionally low. Moreover, it holds a clear advantage over the optimal values of other algorithms like POA (416.63), This demonstrates MIPOA’s potential to discover superior paths, achieving path costs approaching theoretical optimality in certain runs. Combining the “mean,” “max,” and “min” metrics reveals that MIPOA not only achieves lower average path costs and greater algorithmic stability but also demonstrates superior worst-case performance and robustness compared to other algorithms. Additionally, it possesses the capability to discover optimal paths, enabling it to plan lower-cost flight paths more efficiently and stably than other comparison algorithms in path planning tasks.

**Figure 8:**
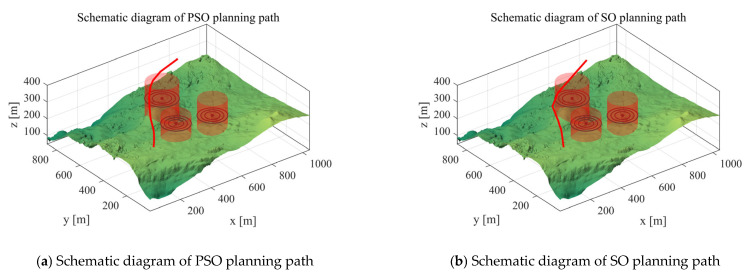

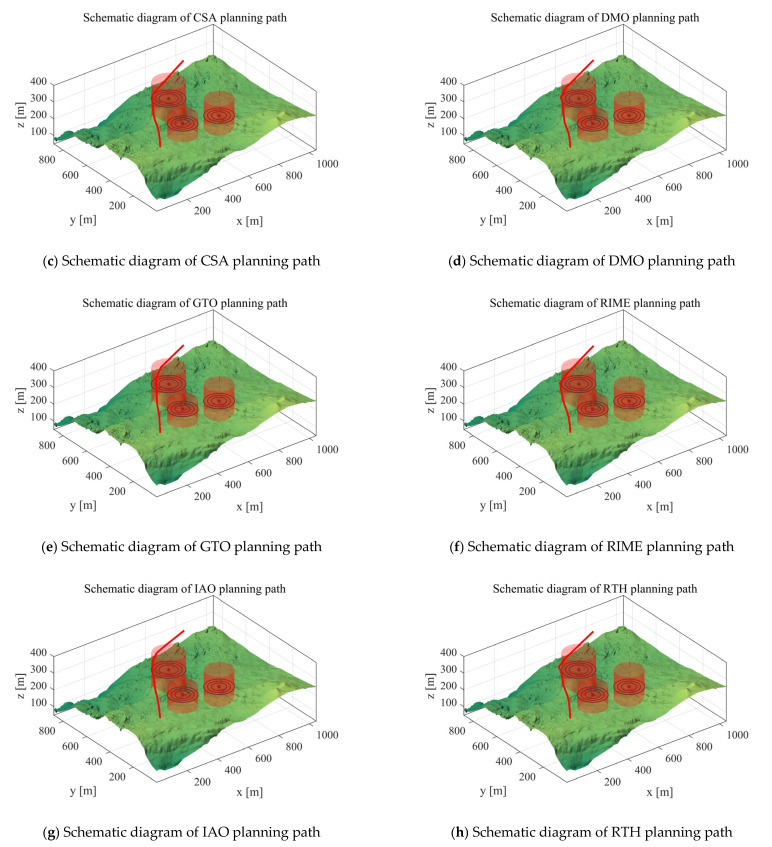

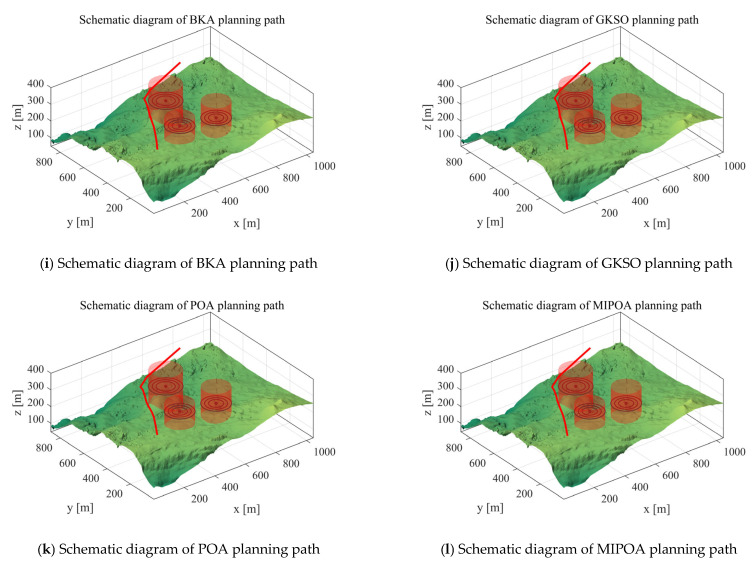
Scenario 2 Schematic diagram of path planning.

*Scenario 2*: We utilized three cylindrical obstacles to mimic real-world hazards and adopted simulation experiments to verify the UAV path planning performance of the MIPOA under two cylindrical threat scenarios. In the experimental configuration, the UAV’s starting position was set to [100, 100, 150], the destination to [800, 800, 150], and the number of waypoints was set as 10. The total path costs are presented in Table 5: “Mean” stands for the average value of 30 independent operation runs, “Max” refers to the highest cost recorded across the 30 algorithm runs, and “Min” represents the optimal cost achieved in the 30 runs. Figure 8 displays the paths planned for the UAV by each algorithm in Scenario 2. The red line indicates the flight path planned for the UAV.

Based on the tabulated data, MIDOA demonstrates overall superior path planning performance compared to the benchmark algorithms PSO, SO, CSA, DMO, GTO, RIME, SAO, ED, GKSO, IGWO, and DOA. Regarding the “mean” metric reflecting average algorithmic performance, MIDOA achieved a mean of 415.92—significantly lower than the 452.38 recorded by the classical PSO algorithm and also below other algorithms like DOA (419.58). This indicates that across 30 independent runs, MIDOA consistently delivered lower average path costs, demonstrating superior algorithmic stability and overall optimization capability. Regarding the “max” metric, which represents the upper limit of an algorithm’s worst-case performance, MIDOA achieved the lowest maximum value among all algorithms at 416.36. In contrast, ED reached a maximum of 435.04, while PSO reached as high as 496.96. Both figures significantly exceeded MIDOA’s maximum. Even compared to DOA’s 433.59, MIDOA’s worst-case path cost remained lower, demonstrating the algorithm’s enhanced robustness in complex scenarios and reduced susceptibility to extreme deviations from optimal solutions. Examining the “min (minimum)” metric, which reflects an algorithm’s potential for optimal performance, MIDOA achieved the lowest minimum value of 413.55 among all algorithms, significantly lower than the optimal values of POA (415.08) and CSA (415.21). This demonstrates MIDOA’s outstanding potential to discover superior paths, achieving path costs closer to theoretical optimality in certain runs. Combining the “mean,” “max,” and “min” metrics reveals that MIDOA achieves lower average path costs and greater algorithmic stability. Its worst-case performance surpasses other algorithms while offering superior robustness. Concurrently, it demonstrates exceptional capability in identifying optimal paths. In path planning tasks, MIDOA efficiently and stably generates lower-cost flight paths compared to other benchmark algorithms.

**Figure 9:**
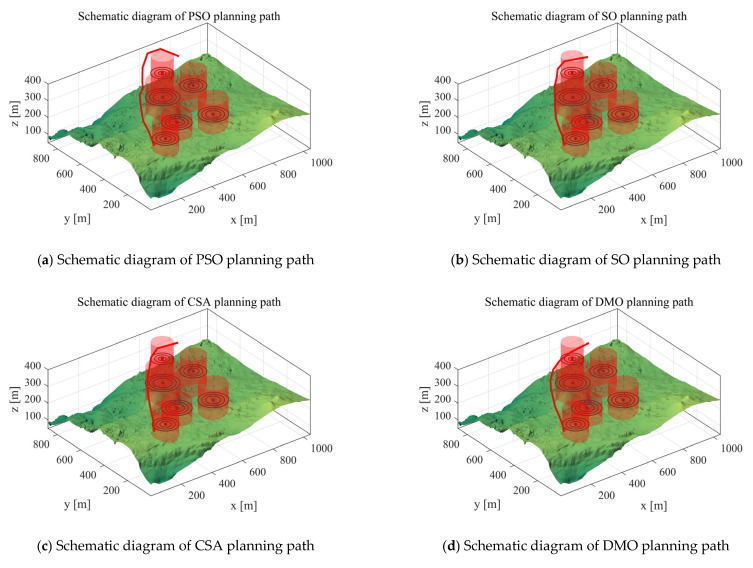

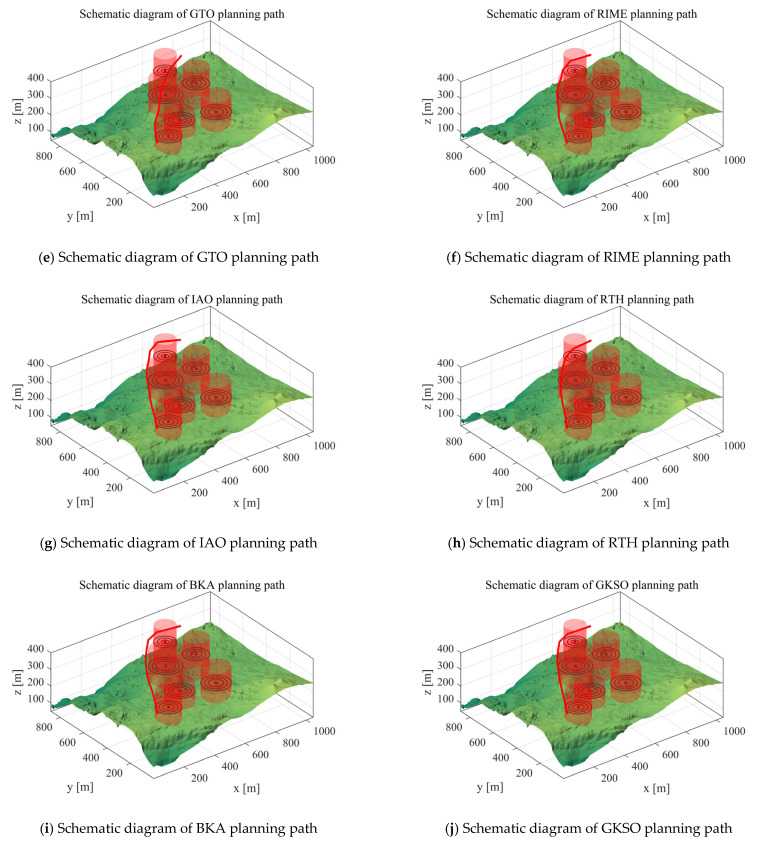

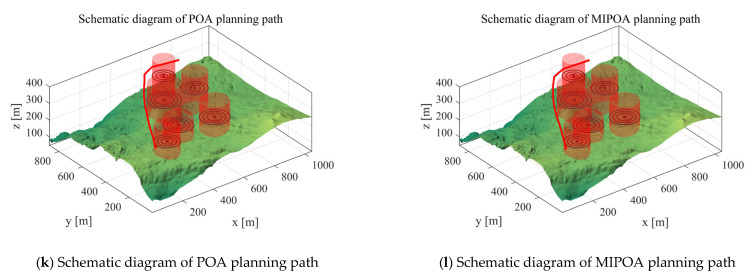
Scenario 3 Schematic diagram of path planning.

*Scenario 3*: We utilized six cylindrical obstacles to mimic real-world hazards and adopted simulation experiments to verify the UAV path planning performance of the MIPOA under two cylindrical threat scenarios. In the experimental configuration, the UAV’s starting position was set to [100, 100, 150], the destination to [800, 800, 150], and the number of waypoints was set as 10. The total path costs are presented in Table 6: “Mean” stands for the average value of 30 independent operation runs, “Max” refers to the highest cost recorded across the 30 algorithm runs, and “Min” represents the optimal cost achieved in the 30 runs. Figure 9 displays the paths planned for the UAV by each algorithm in Scenario 3. The red line indicates the flight path planned for the UAV.

As shown in the table data, MIDOA demonstrates overall superior path planning performance compared to other algorithms such as PSO, SO, CSA, DMO, GTO, RIME, SAO, ED, GKSO, IGWO, and DOA. In the “mean” metric reflecting average performance, MIDOA achieves a mean value of 433.61, significantly lower than other algorithms (e.g., PSO at 529.43, DMO at 459.28, etc.). For the “max” metric, MIDOA’s maximum value of 434.58 is the lowest among all algorithms, far outperforming others (e.g., PSO at 745.03). Regarding the “min” metric, MIDOA’s minimum value of 423.28 also remains at a relatively low level. Overall, MIDOA demonstrates greater efficiency and stability in planning lower-cost flight paths.

**Figure 10:**
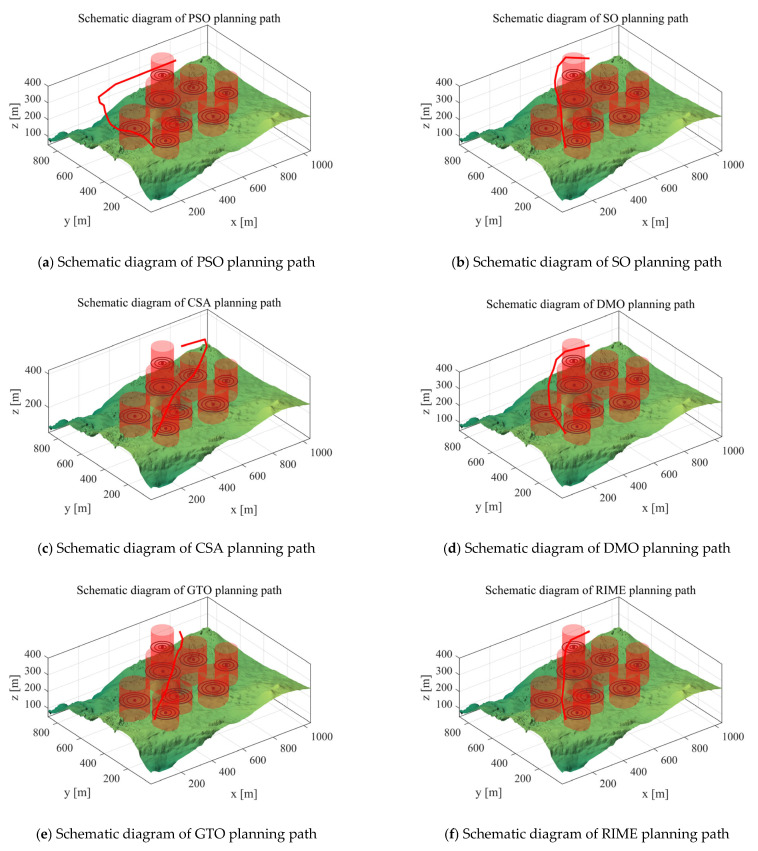

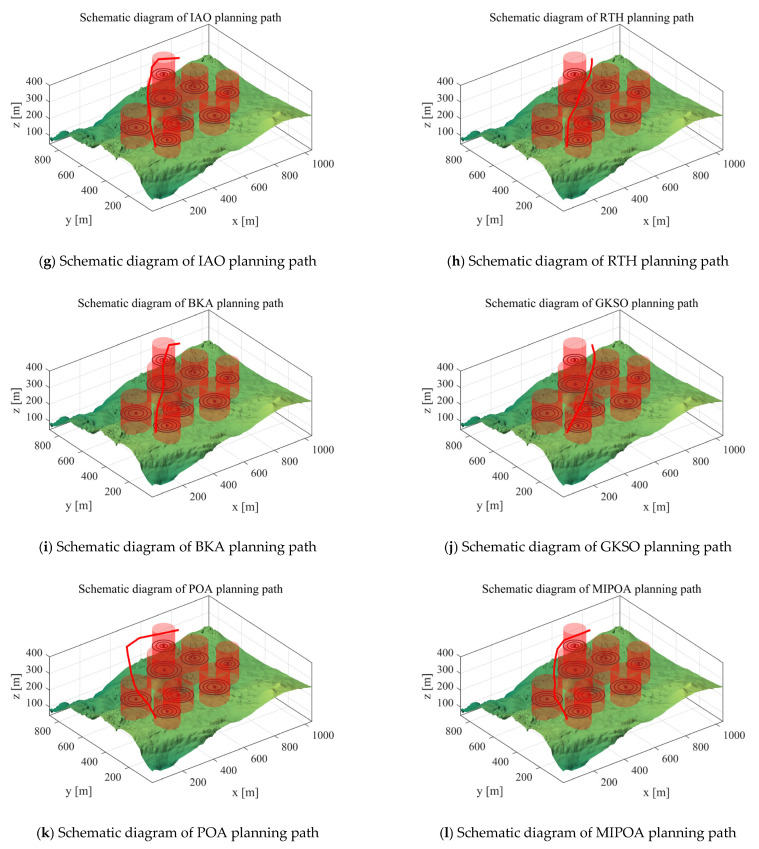
Scenario 4 Schematic diagram of path planning.

*Scenario 4*: We utilized eight cylindrical obstacles to mimic real-world hazards and adopted simulation experiments to verify the UAV path planning performance of the MIPOA under two cylindrical threat scenarios. In the experimental configuration, the UAV’s starting position was set to [100, 100, 150], the destination to [800, 800, 150], and the number of waypoints was set as 10. The total path costs are presented in Table 7: “Mean” stands for the average value of 30 independent operation runs, “Max” refers to the highest cost recorded across the 30 algorithm runs, and “Min” represents the optimal cost achieved in the 30 runs. Figure 10 displays the paths planned for the UAV by each algorithm in Scenario 4. The red line indicates the flight path planned for the UAV.

From the tabular data, MIDOA significantly outperforms PSO, SO, CSA, DMO, GTO, RIME, SAO, ED, GKSO, IGWO, DOA and other comparison algorithms in terms of path planning performance. In the “mean” indicator that reflects the average performance of the algorithms, the mean value of MIDOA is 433.14, which is much lower than that of other algorithms (e.g., as high as 65535.00 for PSO, 481.01 for SO, etc.), which indicates that MIDOA has a low average cost of paths in many independent runs, and the stability of the algorithm and the overall optimization ability are excellent. This indicates that MIDOA has low average path cost, excellent algorithmic stability and overall optimization ability in many independent runs. Regarding the “max” indicator, the max value of MIDOA is 437.81, which is the lowest among all the algorithms, while the max values of PSO is 65535.00 and GKSO is 656.10, which are much higher than that of MIDOA, indicating that MIDOA has strong robustness in complex scenarios and is not prone to drastic deviation from the optimal solution. This shows that MIDOA is robust in complex scenarios and is not prone to extreme cases that deviate from the optimal solution. As for the “min” indicator, the minimum value of MIDOA is 418.57, which is at a lower level and also shows its potential to find a better path. Combining the three dimensions of “mean”, “max” and “min”, MIDOA is able to plan lower cost flight paths more efficiently and stably.

## 6. Conclusions

In this paper, we propose a multi-strategy improved POA to address global optimization problems and 3D UAV path planning to enhance the quality of the initial population, a hybrid low-discrepancy and heuristic initialization method is proposed. This method ensures uniform coverage of the search space through low-difference sequences, while heuristics enhance the quality of some individuals and prevent futile searches. Subsequently, we proposed a subgroup mean-guided update strategy to accelerate the algorithm’s convergence toward the global optimum. Additionally, we propose a random re-initialization boundary control to enhance the algorithm’s exploration capability, enabling it to effectively avoid local optima. Through global optimization and experiments under four threat scenarios, MIPOA demonstrates superior performance.

## Figures and Tables

**Table 1:** Parameter settings of the comparison algorithms.

Algorithms	Parameter Name	Parameter Value	Population Size	Iter Counts	Reference
PSO	Vmax , wMax , wMin , c1 , c2	6, 0.9, 0.6, 2, 2	50	1000	[27]
SO	c1 , c2 , c3	0.5, 0.5, 2	50	1000	[28]
CSA	v, rho, gamma, alpha	0.1, 1.0, 2.0, 4.0	50	1000	[29]
DMO	peep	2	50	1000	[30]
GTO	p, Beta, w	0.03, 3, 0.8	50	1000	[31]
RIME	W	5	50	1000	[32]
IAO	tt	0.5	50	1000	[33]
RTH	A, R0, r	15, 0.5, 1.5	50	1000	[34]
BKA	p	0.9	50	1000	[35]
GKSO	$h\left(1\right)$	0.1	50	1000	[36]
POA	I	2	50	1000	[22]

**Table 2:** Population Diversity Index of POA and MIPOA.

Function	POA	MIPOA	Function	POA	MIPOA
F1	4433	4463	F16	4678	5275
F2	4483	4545	F17	4555	4766
F3	4434	4891	F18	4434	4718
F4	4662	5462	F19	4433	4588
F5	4497	4669	F20	4535	4672
F6	4500	5307	F21	4476	5496
F7	4442	4658	F22	4433	4774
F8	4433	4750	F23	4817	4885
F9	4433	4889	F24	4431	4838
F10	4435	4437	F25	4451	4817
F11	4750	5198	F26	4452	4505
F12	4433	4534	F27	4433	5412
F13	4433	4673	F28	4440	4688
F14	4596	4788	F29	4513	5138
F15	4433	4889	F30	4532	4572

**Table 3:** The ranking of different algorithms on CEC2017.

Suites	CEC2017					
Dimensions	10		30		50	
Algorithms	*M.R*	*T.R*	*M.R*	*T.R*	*M.R*	*T.R*
PSO	8.97	11	6.27	8	5.13	5
SO	6.07	5	5.10	3	5.27	6
CSA	5.83	3	5.57	7	5.73	7
DMO	6.90	8	8.07	9	8.37	9
GTO	6.87	6	5.53	6	5.73	8
RIME	6.87	7	5.30	4	4.87	4
IAO	2.53	2	8.40	10	9.87	11
RTH	8.30	9	5.37	5	4.63	3
BKA	8.53	10	10.10	11	9.17	10
GKSO	5.90	4	4.77	2	4.50	2
POA	8.97	12	10.23	12	10.40	12
MIPOA	**2.27**	**1**	**3.30**	**1**	**4.33**	**1**

**Table 4:** Experimental results of path planning for each algorithm.

Algorithm	PSO	SO	CSA	DMO	GTO	RIME	IAO	RTH	BKA	GKSO	POA	MIPOA
mean	455.47	416.46	418.66	426.14	417.06	418.67	422.64	422.47	418.89	417.70	419.11	416.01
max	501.23	418.27	429.04	430.93	421.85	424.53	427.74	458.59	424.55	420.83	424.53	416.34
min	426.22	414.70	415.11	420.52	414.49	415.57	414.74	415.87	415.87	415.82	416.63	414.90

**Table 5:** Experimental results of path planning for each algorithm.

Algorithm	PSO	SO	CSA	DMO	GTO	RIME	SAO	ED	GKSO	IGWO	DOA	MIDOA
mean	452.38	416.87	418.49	425.10	417.43	419.51	422.75	418.01	421.11	417.56	419.58	415.92
max	496.96	436.36	426.18	430.86	428.86	430.35	428.45	435.04	442.39	419.19	433.59	416.36
min	424.50	415.08	415.21	421.46	414.48	417.02	414.64	415.04	416.16	416.32	417.19	413.55

**Table 6:** Experimental results of path planning for each algorithm.

Algorithm	PSO	SO	CSA	DMO	GTO	RIME	SAO	ED	GKSO	IGWO	DOA	MIDOA
mean	529.43	455.54	451.09	459.28	442.88	453.12	448.24	439.91	450.59	436.22	446.35	433.61
max	745.03	495.73	493.30	469.72	460.18	478.94	471.14	474.17	593.09	467.04	524.56	434.58
min	487.58	434.39	436.75	447.71	433.33	434.32	437.42	433.44	435.91	417.19	437.49	423.28

**Table 7:** Experimental results of path planning for each algorithm.

Algorithm	PSO	SO	CSA	DMO	GTO	RIME	SAO	ED	GKSO	IGWO	DOA	MIDOA
mean	65535.00	481.01	521.68	498.37	472.38	469.24	464.25	496.19	508.45	442.62	488.93	433.14
max	65535.00	546.53	665.56	515.37	553.57	506.37	507.86	598.17	656.10	491.53	555.48	437.81
min	544.66	450.89	452.59	473.78	418.52	434.33	426.45	425.36	441.38	418.16	445.64	418.57

## Data Availability

Data are contained within the article.

## Appendix A

**Table A1:** Descriptions of CEC-2017 benchmark test functions.

No.	Functions	Search Range	Dim	f_min_
Unimodal functions				
F1	Shifted and Rotated Bent Cigar Function	[−100, 100]	30/50/100	100
F3	Shifted and Rotated Zakharov Function	[−100, 100]	30/50/100	300
Simple multimodal functions				
F4	Shifted and Rotated Rosenbrock’s Function	[−100, 100]	30/50/100	400
F5	Shifted and Rotated Rastrigin’s Function	[−100, 100]	30/50/100	500
F6	Shifted and Rotated Expanded Scaffer’s F6 Function	[−100, 100]	30/50/100	600
F7	Shifted and Rotated Lunacek Bi_Rastrigin’s Function	[−100, 100]	30/50/100	700
F8	Shifted and Rotated Non-Continuous Rastrigin’s Function	[−100, 100]	30/50/100	800
F9	Shifted and Rotated Levy Function	[−100, 100]	30/50/100	900
F10	Shifted and Rotated Schwefel’s Function	[−100, 100]	30/50/100	1000
Hybrid functions				
F11	Hybrid Function 1 (N = 3)	[−100, 100]	30/50/100	1100
F12	Hybrid Function 2 (N = 3)	[−100, 100]	30/50/100	1200
F13	Hybrid Function 3 (N = 3)	[−100,100]	30/50/100	1300
F14	Hybrid Function 4 (N = 4)	[−100, 100]	30/50/100	1400
F15	Hybrid Function 5 (N = 4)	[−100, 100]	30/50/100	1500
F16	Hybrid Function 6 (N = 4)	[−100, 100]	30/50/100	1600
F17	Hybrid Function 6 (N = 5)	[−100, 100]	30/50/100	1700
F18	Hybrid Function 6 (N = 5)	[−100, 100]	30/50/100	1800
F19	Hybrid Function 6 (N = 5)	[−100, 100]	30/50/100	1900
F20	Hybrid Function 6 (N = 6)	[−100, 100]	30/50/100	2000
Composition functions				
F21	Composition Function 1 (N = 3)	[−100, 100]	30/50/100	2100
F22	Composition Function 2 (N = 3)	[−100, 100]	30/50/100	2200
F23	Composition Function 3 (N = 4)	[−100, 100]	30/50/100	2300
F24	Composition Function 4 (N = 4)	[−100, 100]	30/50/100	2400
F25	Composition Function 5 (N = 5)	[−100, 100]	30/50/100	2500
F26	Composition Function 6 (N = 5)	[−100, 100]	30/50/100	2600
F27	Composition Function 7 (N = 6)	[−100, 100]	30/50/100	2700
F28	Composition Function 8 (N = 6)	[−100, 100]	30/50/100	2800
F29	Composition Function 9 (N = 3)	[−100, 100]	30/50/100	2900
F30	Composition Function 10 (N = 3)	[−100, 100]	30/50/100	3000

**Table A2:** Results of various algorithms tested on the CEC 2017 benchmark (dim = 10).

ID	Metric	PSO	SO	CSA	DMO	GTO	RIME	IAO	RTH	BKA	GKSO	POA	MIPOA
F1	mean	2.3856 × 10^3^	1.7081 × 10^3^	2.0122 × 10^3^	9.2414 × 10^2^	1.2223 × 10^3^	6.0571 × 10^3^	1.0000 × 10^2^	1.3941 × 10^2^	2.7736 × 10^8^	2.4571 × 10^3^	7.6992 × 10^7^	1.0171 × 10^2^
	std	2.9881 × 10^3^	2.2529 × 10^3^	1.8498 × 10^3^	8.4574 × 10^2^	1.1467 × 10^3^	3.7123 × 10^3^	1.6480 × 10^−14^	1.0087 × 10^2^	9.0851 × 10^8^	2.3363 × 10^3^	2.9482 × 10^8^	2.0654
F2	mean	2.0000 × 10^2^	3.4207 × 10^3^	1.9186 × 10^3^	3.0307 × 10^2^	5.4473 × 10^2^	2.2760 × 10^2^	2.0017 × 10^2^	2.0853 × 10^2^	1.3089 × 10^5^	2.0003 × 10^2^	1.9135 × 10^4^	2.0000 × 10^2^
	std	0.0000	9.5181 × 10^3^	2.9106 × 10^3^	1.9163 × 10^2^	1.7877 × 10^3^	3.2860 × 10^1^	3.7905 × 10^−1^	2.8470 × 10^1^	4.6116 × 10^5^	1.8257 × 10^−1^	7.8378 × 10^4^	0.0000
F3	mean	3.0000 × 10^2^	3.0000 × 10^2^	3.0000 × 10^2^	3.3189 × 10^2^	3.0000 × 10^2^	3.0005 × 10^2^	3.0000 × 10^2^	3.0000 × 10^2^	3.0073 × 10^2^	3.0000 × 10^2^	4.9433 × 10^2^	3.0000 × 10^2^
	std	1.6548 × 10^−4^	1.4877 × 10^−3^	9.1651 × 10^−7^	3.1247 × 10^1^	4.7170 × 10^−13^	3.5916 × 10^−2^	1.8283 × 10^−14^	4.7206 × 10^−14^	1.7602	6.3448 × 10^−13^	4.7099 × 10^2^	3.4059 × 10^−6^
F4	mean	4.0359 × 10^2^	4.0432 × 10^2^	4.0464 × 10^2^	4.0483 × 10^2^	4.0005 × 10^2^	4.0515 × 10^2^	4.0000 × 10^2^	4.0000 × 10^2^	4.1021 × 10^2^	4.0000 × 10^2^	4.1778 × 10^2^	4.0001 × 10^2^
	std	1.3110	1.0064	2.3497	2.7069 × 10^−1^	1.4248 × 10^−1^	1.4139	6.2040 × 10^−10^	1.4749 × 10^−12^	2.8373 × 10^1^	4.7864 × 10^−3^	2.3768 × 10^1^	7.8635 × 10^−3^
F5	mean	5.3479 × 10^2^	5.0860 × 10^2^	5.0942 × 10^2^	5.2225 × 10^2^	5.2223 × 10^2^	5.1093 × 10^2^	5.0981 × 10^2^	5.3177 × 10^2^	5.3176 × 10^2^	5.2564 × 10^2^	5.3744 × 10^2^	5.0993 × 10^2^
	std	1.0602 × 10^1^	3.9774	4.1866	5.0772	1.0736 × 10^1^	4.9328	5.8479	1.3190 × 10^1^	1.7124 × 10^1^	1.3279 × 10^1^	1.4698 × 10^1^	2.0623
F6	mean	6.0614 × 10^2^	6.0006 × 10^2^	6.0156 × 10^2^	6.0000 × 10^2^	6.0346 × 10^2^	6.0008 × 10^2^	6.0399 × 10^2^	6.0745 × 10^2^	6.2094 × 10^2^	6.0229 × 10^2^	6.1767 × 10^2^	6.0054 × 10^2^
	std	5.4520	9.9274 × 10^−2^	9.2610 × 10^−1^	0.0000	2.9773	5.8481 × 10^−2^	5.8275	6.5398	1.1326 × 10^1^	3.0874	9.4542	2.5883 × 10^−1^
F7	mean	7.2433 × 10^2^	7.1977 × 10^2^	7.2004 × 10^2^	7.3488 × 10^2^	7.4095 × 10^2^	7.2099 × 10^2^	7.2295 × 10^2^	7.5242 × 10^2^	7.5209 × 10^2^	7.3464 × 10^2^	7.6167 × 10^2^	7.2076 × 10^2^
	std	5.5454	3.7734	3.5699	6.0218	1.4057 × 10^1^	5.8978	7.0550	1.7280 × 10^1^	1.5444 × 10^1^	1.1282 × 10^1^	1.5747 × 10^1^	2.7182
F8	mean	8.1847 × 10^2^	8.0907 × 10^2^	8.0750 × 10^2^	8.2290 × 10^2^	8.2371 × 10^2^	8.1224 × 10^2^	8.1014 × 10^2^	8.2186 × 10^2^	8.1673 × 10^2^	8.2083 × 10^2^	8.2257 × 10^2^	8.0982 × 10^2^
	std	6.7008	5.6088	3.6368	4.5489	8.5519	3.8679	5.7331	9.0077	6.5417	8.3118	6.7934	1.5402
F9	mean	9.2323 × 10^2^	9.0022 × 10^2^	9.0353 × 10^2^	9.0000 × 10^2^	9.3929 × 10^2^	9.0001 × 10^2^	9.1354 × 10^2^	9.8679 × 10^2^	1.0731 × 10^3^	9.0148 × 10^2^	1.0338 × 10^3^	9.0008 × 10^2^
	std	8.1361 × 10^1^	2.5522 × 10^−1^	2.7670	0.0000	3.8127 × 10^1^	2.6785 × 10^−2^	3.8641 × 10^1^	8.4907 × 10^1^	8.4032 × 10^1^	2.4557	9.6555 × 10^1^	7.0933 × 10^−2^
F10	mean	1.9044 × 10^3^	1.3797 × 10^3^	1.5890 × 10^3^	2.0561 × 10^3^	1.8259 × 10^3^	1.4082 × 10^3^	1.4571 × 10^3^	1.9452 × 10^3^	1.8022 × 10^3^	1.7535 × 10^3^	1.6075 × 10^3^	1.2400 × 10^3^
	std	2.7179 × 10^2^	3.2223 × 10^2^	2.4007 × 10^2^	1.7430 × 10^2^	2.4684 × 10^2^	1.8170 × 10^2^	1.4921 × 10^2^	2.8712 × 10^2^	2.9015 × 10^2^	2.9005 × 10^2^	2.4731 × 10^2^	9.8284 × 10^1^
F11	mean	1.1380 × 10^3^	1.1068 × 10^3^	1.1227 × 10^3^	1.1041 × 10^3^	1.1217 × 10^3^	1.1103 × 10^3^	1.1022 × 10^3^	1.1411 × 10^3^	1.1289 × 10^3^	1.1170 × 10^3^	1.1386 × 10^3^	1.1046 × 10^3^
	std	2.0918 × 10^1^	4.2264	1.4215 × 10^1^	8.3713 × 10^−1^	1.4995 × 10^1^	4.9238	2.5295	3.0737 × 10^1^	2.3346 × 10^1^	1.9158 × 10^1^	2.6502 × 10^1^	1.4261
F12	mean	1.2873 × 10^4^	1.3038 × 10^4^	8.9658 × 10^3^	9.9579 × 10^4^	9.0081 × 10^3^	1.9553 × 10^4^	1.4451 × 10^3^	3.1185 × 10^3^	1.8248 × 10^4^	1.3108 × 10^4^	5.3067 × 10^4^	1.3427 × 10^3^
	std	7.9436 × 10^3^	9.6121 × 10^3^	8.2015 × 10^3^	6.4623 × 10^4^	9.4741 × 10^3^	1.7285 × 10^4^	1.8848 × 10^2^	1.8207 × 10^3^	1.2934 × 10^4^	1.4147 × 10^4^	1.0782 × 10^5^	6.0159 × 10^1^
F13	mean	8.1942 × 10^3^	7.5436 × 10^3^	1.8136 × 10^3^	2.1975 × 10^3^	1.5307 × 10^3^	9.2592 × 10^3^	1.3045 × 10^3^	1.5467 × 10^3^	1.8087 × 10^3^	1.4932 × 10^3^	2.0884 × 10^3^	1.3082 × 10^3^
	std	6.1294 × 10^3^	7.3509 × 10^3^	3.5239 × 10^2^	5.8766 × 10^2^	2.2925 × 10^2^	9.3058 × 10^3^	3.8096	2.2020 × 10^2^	2.9037 × 10^2^	1.5354 × 10^2^	5.3764 × 10^2^	3.5778
F14	mean	1.6543 × 10^3^	1.4767 × 10^3^	1.4367 × 10^3^	1.4787 × 10^3^	1.4606 × 10^3^	1.8200 × 10^3^	1.4024 × 10^3^	1.4880 × 10^3^	1.4723 × 10^3^	1.4331 × 10^3^	1.4465 × 10^3^	1.4056 × 10^3^
	std	4.5884 × 10^2^	3.5938 × 10^1^	1.2518 × 10^1^	2.0636 × 10^1^	3.1245 × 10^1^	7.3630 × 10^2^	3.9571	4.1668 × 10^1^	2.7623 × 10^1^	1.3177 × 10^1^	1.7135 × 10^1^	2.5043
F15	mean	1.9578 × 10^3^	1.7259 × 10^3^	1.5905 × 10^3^	1.6893 × 10^3^	1.5380 × 10^3^	2.2626 × 10^3^	1.5004 × 10^3^	1.5828 × 10^3^	1.6118 × 10^3^	1.5202 × 10^3^	1.5877 × 10^3^	1.5025 × 10^3^
	std	8.0362 × 10^2^	1.2566 × 10^2^	6.5380 × 10^1^	1.0283 × 10^2^	3.4263 × 10^1^	1.3272 × 10^3^	3.2107 × 10^−1^	6.8018 × 10^1^	2.3747 × 10^2^	1.5937 × 10^1^	5.9432 × 10^1^	7.9964 × 10^−1^
F16	mean	1.8312 × 10^3^	1.6028 × 10^3^	1.6284 × 10^3^	1.6062 × 10^3^	1.6750 × 10^3^	1.7116 × 10^3^	1.6028 × 10^3^	1.7831 × 10^3^	1.7383 × 10^3^	1.6561 × 10^3^	1.7373 × 10^3^	1.6016 × 10^3^
	std	1.3806 × 10^2^	2.5135	3.6288 × 10^1^	3.5341	8.4054 × 10^1^	1.0041 × 10^2^	1.8743	1.4627 × 10^2^	9.2591 × 10^1^	5.8843 × 10^1^	8.4176 × 10^1^	3.2442 × 10^−1^
F17	mean	1.7584 × 10^3^	1.7240 × 10^3^	1.7339 × 10^3^	1.7343 × 10^3^	1.7398 × 10^3^	1.7522 × 10^3^	1.7296 × 10^3^	1.7671 × 10^3^	1.7595 × 10^3^	1.7354 × 10^3^	1.7466 × 10^3^	1.7255 × 10^3^
	std	3.9980 × 10^1^	1.0670 × 10^1^	8.9509	7.0625	1.7130 × 10^1^	4.7848 × 10^1^	7.7925	4.0761 × 10^1^	4.1839 × 10^1^	1.8294 × 10^1^	1.0139 × 10^1^	3.6943
F18	mean	9.4365 × 10^3^	1.3079 × 10^4^	2.0104 × 10^3^	6.2453 × 10^3^	1.9253 × 10^3^	8.8219 × 10^3^	1.8120 × 10^3^	2.5851 × 10^3^	1.9682 × 10^3^	1.9692 × 10^3^	2.4264 × 10^3^	1.8167 × 10^3^
	std	7.0252 × 10^3^	1.2328 × 10^4^	1.9818 × 10^2^	3.1808 × 10^3^	8.7389 × 10^1^	8.3142 × 10^3^	9.6535	2.3981 × 10^3^	1.2263 × 10^2^	1.1552 × 10^2^	1.8765 × 10^3^	6.4960
F19	mean	3.0836 × 10^3^	2.2656 × 10^3^	1.9428 × 10^3^	1.9940 × 10^3^	1.9401 × 10^3^	2.9815 × 10^3^	1.9013 × 10^3^	1.9662 × 10^3^	1.9339 × 10^3^	1.9099 × 10^3^	1.9397 × 10^3^	1.9025 × 10^3^
	std	1.7602 × 10^3^	6.7206 × 10^2^	6.0463 × 10^1^	6.8486 × 10^1^	3.1974 × 10^1^	2.3355 × 10^3^	7.8741 × 10^−1^	3.9985 × 10^1^	2.9913 × 10^1^	7.8082	3.5773 × 10^1^	4.9913 × 10^−1^
F20	mean	2.1030 × 10^3^	2.0178 × 10^3^	2.0290 × 10^3^	2.0006 × 10^3^	2.0494 × 10^3^	2.0148 × 10^3^	2.0205 × 10^3^	2.1460 × 10^3^	2.0682 × 10^3^	2.0306 × 10^3^	2.0552 × 10^3^	2.0215 × 10^3^
	std	6.7392 × 10^1^	1.0731 × 10^1^	9.1290	1.0886	3.1247 × 10^1^	2.3151 × 10^1^	7.0400	6.5394 × 10^1^	3.7634 × 10^1^	1.4306 × 10^1^	2.6692 × 10^1^	3.4791
F21	mean	2.3063 × 10^3^	2.3109 × 10^3^	2.2807 × 10^3^	2.2679 × 10^3^	2.2018 × 10^3^	2.2874 × 10^3^	2.2002 × 10^3^	2.3304 × 10^3^	2.2585 × 10^3^	2.2049 × 10^3^	2.2421 × 10^3^	2.1967 × 10^3^
	std	5.5113 × 10^1^	1.6946 × 10^1^	4.8453 × 10^1^	4.4057 × 10^1^	1.5038	5.2954 × 10^1^	7.5577 × 10^−1^	3.7695 × 10^1^	6.2332 × 10^1^	3.5387 × 10^1^	5.8283 × 10^1^	1.8257 × 10^1^
F22	mean	2.3120 × 10^3^	2.3021 × 10^3^	2.2965 × 10^3^	2.3002 × 10^3^	2.3019 × 10^3^	2.2936 × 10^3^	2.3008 × 10^3^	2.3022 × 10^3^	2.3071 × 10^3^	2.2986 × 10^3^	2.3141 × 10^3^	2.2768 × 10^3^
	std	5.5746 × 10^1^	9.4581 × 10^−1^	1.9579 × 10^1^	2.7318	1.5187 × 10^1^	2.4258 × 10^1^	5.7347 × 10^−1^	1.0909 × 10^1^	6.5675	1.8050 × 10^1^	3.3935 × 10^1^	2.9977 × 10^1^
F23	mean	2.6818 × 10^3^	2.6099 × 10^3^	2.6133 × 10^3^	2.6175 × 10^3^	2.6141 × 10^3^	2.6159 × 10^3^	2.6115 × 10^3^	2.6289 × 10^3^	2.6359 × 10^3^	2.6293 × 10^3^	2.6431 × 10^3^	2.5799 × 10^3^
	std	3.3427 × 10^1^	3.8647	5.4417	3.9944	6.0737 × 10^1^	5.6617	6.0457	1.5901 × 10^1^	1.9003 × 10^1^	1.1956 × 10^1^	1.6728 × 10^1^	9.4920 × 10^1^
F24	mean	2.7351 × 10^3^	2.7396 × 10^3^	2.7325 × 10^3^	2.7043 × 10^3^	2.7013 × 10^3^	2.7315 × 10^3^	2.5602 × 10^3^	2.7503 × 10^3^	2.7398 × 10^3^	2.6891 × 10^3^	2.6055 × 10^3^	2.4924 × 10^3^
	std	1.2765 × 10^2^	6.7077	4.4081 × 10^1^	4.7028 × 10^1^	1.0280 × 10^2^	6.3243 × 10^1^	1.0751 × 10^2^	4.8868 × 10^1^	6.6543 × 10^1^	1.1667 × 10^2^	1.2746 × 10^2^	2.3448 × 10^1^
F25	mean	2.9185 × 10^3^	2.9232 × 10^3^	2.9222 × 10^3^	2.9222 × 10^3^	2.9346 × 10^3^	2.9243 × 10^3^	2.9149 × 10^3^	2.9173 × 10^3^	2.9031 × 10^3^	2.9300 × 10^3^	2.9132 × 10^3^	2.8879 × 10^3^
	std	6.5997 × 10^1^	2.3744 × 10^1^	2.3409 × 10^1^	1.9438 × 10^1^	2.2096 × 10^1^	2.4286 × 10^1^	2.2190 × 10^1^	6.4741 × 10^1^	8.8304 × 10^1^	2.2608 × 10^1^	2.3790 × 10^1^	5.4167 × 10^1^
F26	mean	3.1352 × 10^3^	2.9334 × 10^3^	2.9354 × 10^3^	2.8739 × 10^3^	2.9601 × 10^3^	3.0111 × 10^3^	2.8667 × 10^3^	3.1586 × 10^3^	3.0089 × 10^3^	2.9109 × 10^3^	3.0251 × 10^3^	2.6404 × 10^3^
	std	3.5165 × 10^2^	1.6621 × 10^2^	5.1280 × 10^1^	4.4012 × 10^1^	7.5092 × 10^1^	3.2461 × 10^2^	9.2227 × 10^1^	3.6605 × 10^2^	2.5939 × 10^2^	8.2133 × 10^1^	3.0268 × 10^2^	8.1179 × 10^1^
F27	mean	3.1443 × 10^3^	3.0905 × 10^3^	3.0936 × 10^3^	3.0898 × 10^3^	3.0948 × 10^3^	3.0947 × 10^3^	3.0908 × 10^3^	3.1055 × 10^3^	3.1009 × 10^3^	3.0996 × 10^3^	3.1028 × 10^3^	3.0714 × 10^3^
	std	5.1226 × 10^1^	1.1505	2.1731	4.0542 × 10^−1^	3.9185	4.0787	2.7376	2.0760 × 10^1^	1.6145 × 10^1^	1.6508 × 10^1^	2.3316 × 10^1^	1.5662 × 10^−1^
F28	mean	3.1593 × 10^3^	3.3700 × 10^3^	3.3831 × 10^3^	3.1948 × 10^3^	3.2300 × 10^3^	3.2441 × 10^3^	3.1558 × 10^3^	3.3667 × 10^3^	3.2846 × 10^3^	3.2949 × 10^3^	3.2092 × 10^3^	3.0816 × 10^3^
	std	3.9504 × 10^1^	8.1749 × 10^1^	1.3219 × 10^2^	3.3446 × 10^1^	1.4884 × 10^2^	1.2724 × 10^2^	1.0617 × 10^2^	1.9725 × 10^2^	1.3828 × 10^2^	1.4833 × 10^2^	9.7186 × 10^1^	7.0302 × 10^1^
F29	mean	3.2290 × 10^3^	3.1639 × 10^3^	3.1630 × 10^3^	3.1908 × 10^3^	3.1941 × 10^3^	3.1768 × 10^3^	3.1483 × 10^3^	3.2723 × 10^3^	3.2226 × 10^3^	3.1888 × 10^3^	3.2014 × 10^3^	3.1439 × 10^3^
	std	4.6895 × 10^1^	1.6912 × 10^1^	2.8535 × 10^1^	1.4277 × 10^1^	5.0765 × 10^1^	3.3359 × 10^1^	1.2537 × 10^1^	7.3769 × 10^1^	4.7467 × 10^1^	4.0145 × 10^1^	3.5853 × 10^1^	4.0749
F30	mean	1.7760 × 10^4^	2.1272 × 10^5^	1.4345 × 10^5^	4.8851 × 10^4^	8.9597 × 10^5^	2.5868 × 10^5^	4.8011 × 10^4^	4.3742 × 10^5^	2.6654 × 10^5^	2.5003 × 10^5^	1.2044 × 10^5^	3.2026 × 10^3^
	std	1.3874 × 10^4^	3.8309 × 10^5^	2.3821 × 10^5^	3.3600 × 10^4^	1.1484 × 10^6^	4.2288 × 10^5^	1.7850 × 10^5^	6.0427 × 10^5^	6.2004 × 10^5^	3.8000 × 10^5^	3.4127 × 10^5^	2.8759 × 10^−1^

**Table A3:** Results of various algorithms tested on the CEC 2017 benchmark (dim = 30).

ID	Metric	PSO	SO	CSA	DMO	GTO	RIME	IAO	RTH	BKA	GKSO	POA	MIPOA
F1	mean	2.3887 × 10^5^	4.1212 × 10^6^	5.3715 × 10^4^	1.8116 × 10^6^	5.5840 × 10^3^	3.3394 × 10^5^	2.2697 × 10^10^	2.4027 × 10^3^	6.3356 × 10^9^	3.0529 × 10^3^	1.2952 × 10^10^	5.8125 × 10^7^
	std	2.0175 × 10^5^	6.9561 × 10^6^	1.4589 × 10^5^	2.4935 × 10^6^	6.2617 × 10^3^	1.5203 × 10^5^	7.8430 × 10^9^	2.4311 × 10^3^	9.6751 × 10^9^	3.0859 × 10^3^	5.2422 × 10^9^	3.2562 × 10^7^
F2	mean	8.8878 × 10^9^	3.9737 × 10^21^	5.7904 × 10^21^	2.9900 × 10^31^	9.5564 × 10^19^	1.3117 × 10^13^	1.1162 × 10^29^	2.4334 × 10^16^	1.7475 × 10^35^	2.4919 × 10^12^	3.2294 × 10^32^	3.3591 × 10^15^
	std	1.5201 × 10^10^	1.1207 × 10^22^	2.6129 × 10^22^	7.4630 × 10^31^	3.4676 × 10^20^	3.0103 × 10^13^	3.2659 × 10^29^	1.3319 × 10^17^	9.5713 × 10^35^	7.2226 × 10^12^	1.3240 × 10^33^	4.3542 × 10^15^
F3	mean	5.8438 × 10^3^	5.8222 × 10^4^	3.3874 × 10^4^	1.2183 × 10^5^	9.3618 × 10^2^	6.2407 × 10^3^	3.9258 × 10^4^	3.0000 × 10^2^	1.1287 × 10^4^	3.0686 × 10^2^	3.2265 × 10^4^	1.3446 × 10^3^
	std	2.8840 × 10^3^	1.1117 × 10^4^	9.9291 × 10^3^	1.6997 × 10^4^	6.7845 × 10^2^	3.1434 × 10^3^	1.1985 × 10^4^	3.2956 × 10^−9^	8.8805 × 10^3^	8.7978	7.5831 × 10^3^	4.1728 × 10^2^
F4	mean	4.6617 × 10^2^	5.0048 × 10^2^	5.1651 × 10^2^	5.1924 × 10^2^	4.9865 × 10^2^	5.0876 × 10^2^	3.8591 × 10^3^	4.1478 × 10^2^	1.1379 × 10^3^	5.0332 × 10^2^	2.0709 × 10^3^	4.9676 × 10^2^
	std	2.8965 × 10^1^	7.4753	3.5997 × 10^1^	1.4727 × 10^1^	2.9284 × 10^1^	1.7870 × 10^1^	1.9037 × 10^3^	2.3442 × 10^1^	1.9227 × 10^3^	2.0892 × 10^1^	1.3256 × 10^3^	1.2709 × 10^1^
F5	mean	6.7086 × 10^2^	5.6521 × 10^2^	5.9546 × 10^2^	7.1824 × 10^2^	6.9096 × 10^2^	5.8907 × 10^2^	7.3458 × 10^2^	6.8006 × 10^2^	7.4552 × 10^2^	6.7585 × 10^2^	7.5945 × 10^2^	6.5495 × 10^2^
	std	2.9210 × 10^1^	2.3066 × 10^1^	2.0244 × 10^1^	1.2756 × 10^1^	4.4679 × 10^1^	1.9869 × 10^1^	3.7477 × 10^1^	4.6841 × 10^1^	4.2469 × 10^1^	4.7296 × 10^1^	3.2677 × 10^1^	1.1852 × 10^1^
F6	mean	6.3913 × 10^2^	6.0201 × 10^2^	6.2206 × 10^2^	6.0049 × 10^2^	6.3917 × 10^2^	6.0311 × 10^2^	6.5288 × 10^2^	6.3864 × 10^2^	6.6084 × 10^2^	6.3738 × 10^2^	6.5892 × 10^2^	6.3431 × 10^2^
	std	7.5776	6.2737 × 10^−1^	3.8666	1.0403 × 10^−1^	6.8787	2.4983	9.5613	8.7310	6.6730	8.3636	8.3218	3.9429
F7	mean	8.4865 × 10^2^	7.8068 × 10^2^	9.0196 × 10^2^	9.6384 × 10^2^	1.0353 × 10^3^	8.2806 × 10^2^	1.1142 × 10^3^	1.0848 × 10^3^	1.1631 × 10^3^	9.4314 × 10^2^	1.2456 × 10^3^	9.4878 × 10^2^
	std	4.0650 × 10^1^	1.3518 × 10^1^	3.9050 × 10^1^	1.2216 × 10^1^	8.3044 × 10^1^	2.8262 × 10^1^	7.8197 × 10^1^	1.0283 × 10^2^	7.6104 × 10^1^	7.8029 × 10^1^	8.1429 × 10^1^	3.5823 × 10^1^
F8	mean	9.1568 × 10^2^	8.8393 × 10^2^	8.7566 × 10^2^	1.0201 × 10^3^	9.5238 × 10^2^	8.8069 × 10^2^	9.9803 × 10^2^	9.3997 × 10^2^	9.6763 × 10^2^	9.3860 × 10^2^	9.8721 × 10^2^	9.1897 × 10^2^
	std	2.0356 × 10^1^	4.0500 × 10^1^	1.5559 × 10^1^	1.6290 × 10^1^	2.8459 × 10^1^	1.7130 × 10^1^	2.6693 × 10^1^	2.5179 × 10^1^	3.2740 × 10^1^	2.6754 × 10^1^	3.2120 × 10^1^	1.0338 × 10^1^
F9	mean	4.3171 × 10^3^	9.4265 × 10^2^	1.7217 × 10^3^	1.0651 × 10^3^	3.9133 × 10^3^	1.7294 × 10^3^	5.0062 × 10^3^	4.2688 × 10^3^	5.2233 × 10^3^	3.0113 × 10^3^	5.2922 × 10^3^	2.7618 × 10^3^
	std	1.1451 × 10^3^	1.8068 × 10^1^	3.5158 × 10^2^	4.6318 × 10^1^	1.0492 × 10^3^	9.8103 × 10^2^	1.2513 × 10^3^	8.4554 × 10^2^	1.5914 × 10^3^	7.3000 × 10^2^	6.9489 × 10^2^	3.4390 × 10^2^
F10	mean	4.7853 × 10^3^	7.2348 × 10^3^	5.0804 × 10^3^	8.3930 × 10^3^	5.4580 × 10^3^	4.2668 × 10^3^	5.4988 × 10^3^	5.0910 × 10^3^	5.3249 × 10^3^	4.6522 × 10^3^	4.9332 × 10^3^	3.8602 × 10^3^
	std	6.0241 × 10^2^	1.1399 × 10^3^	6.8059 × 10^2^	2.4191 × 10^2^	6.9727 × 10^2^	8.1309 × 10^2^	4.6110 × 10^2^	4.7006 × 10^2^	1.1317 × 10^3^	6.4247 × 10^2^	4.8405 × 10^2^	2.5247 × 10^2^
F11	mean	1.2108 × 10^3^	1.2687 × 10^3^	1.2831 × 10^3^	1.3159 × 10^3^	1.2277 × 10^3^	1.2828 × 10^3^	1.6494 × 10^3^	1.2323 × 10^3^	1.4747 × 10^3^	1.2129 × 10^3^	1.8163 × 10^3^	1.2266 × 10^3^
	std	3.5856 × 10^1^	5.9229 × 10^1^	5.5672 × 10^1^	2.4824 × 10^1^	4.4392 × 10^1^	6.1624 × 10^1^	2.9170 × 10^2^	5.6626 × 10^1^	4.8748 × 10^2^	4.4994 × 10^1^	5.8934 × 10^2^	2.0263 × 10^1^
F12	mean	1.3674 × 10^6^	4.6405 × 10^5^	2.0060 × 10^7^	1.1783 × 10^7^	2.6780 × 10^5^	6.4665 × 10^6^	3.0142 × 10^8^	2.3418 × 10^4^	7.3454 × 10^8^	1.4641 × 10^5^	7.3355 × 10^8^	4.1859 × 10^4^
	std	1.0342 × 10^6^	6.7269 × 10^5^	1.7733 × 10^7^	4.9616 × 10^6^	3.5112 × 10^5^	4.6376 × 10^6^	4.2146 × 10^8^	9.9337 × 10^3^	1.5460 × 10^9^	1.4323 × 10^5^	9.7443 × 10^8^	2.0009 × 10^4^
F13	mean	1.7251 × 10^4^	3.3747 × 10^4^	8.9635 × 10^4^	1.2941 × 10^4^	1.8523 × 10^4^	7.5503 × 10^4^	2.0894 × 10^4^	2.0063 × 10^4^	6.7048 × 10^7^	1.2284 × 10^4^	9.9304 × 10^6^	1.6918 × 10^4^
	std	1.5792 × 10^4^	3.1224 × 10^4^	5.4861 × 10^4^	6.6435 × 10^3^	2.1306 × 10^4^	8.2531 × 10^4^	8.6800 × 10^3^	2.1076 × 10^4^	2.5169 × 10^8^	1.1789 × 10^4^	4.6597 × 10^7^	5.7752 × 10^3^
F14	mean	2.5616 × 10^4^	2.7050 × 10^4^	2.7686 × 10^3^	6.2063 × 10^4^	2.3805 × 10^3^	3.4112 × 10^4^	1.5248 × 10^3^	1.8808 × 10^3^	6.1431 × 10^3^	2.4914 × 10^3^	1.0540 × 10^4^	1.5340 × 10^3^
	std	2.8730 × 10^4^	3.7991 × 10^4^	2.2196 × 10^3^	3.6532 × 10^4^	1.4626 × 10^3^	2.9479 × 10^4^	3.3099 × 10^1^	4.5072 × 10^2^	9.2527 × 10^3^	1.1878 × 10^3^	1.9051 × 10^4^	1.4269 × 10^1^
F15	mean	1.0135 × 10^4^	1.0427 × 10^4^	1.9123 × 10^4^	4.4067 × 10^3^	7.8583 × 10^3^	1.4598 × 10^4^	3.3242 × 10^3^	1.0190 × 10^4^	2.9930 × 10^4^	8.6824 × 10^3^	4.2329 × 10^4^	2.5729 × 10^3^
	std	8.3846 × 10^3^	1.4153 × 10^4^	1.3021 × 10^4^	4.0265 × 10^3^	8.4136 × 10^3^	1.2569 × 10^4^	1.3391 × 10^3^	1.0183 × 10^4^	1.7070 × 10^4^	1.1697 × 10^4^	4.7107 × 10^4^	2.7381 × 10^2^
F16	mean	2.7400 × 10^3^	2.5257 × 10^3^	2.3963 × 10^3^	3.2242 × 10^3^	2.6644 × 10^3^	2.5929 × 10^3^	2.4643 × 10^3^	2.8506 × 10^3^	3.1579 × 10^3^	2.6366 × 10^3^	2.9138 × 10^3^	2.2002 × 10^3^
	std	3.0491 × 10^2^	4.4204 × 10^2^	2.1649 × 10^2^	1.8173 × 10^2^	2.6823 × 10^2^	2.0684 × 10^2^	1.7104 × 10^2^	3.6231 × 10^2^	7.7074 × 10^2^	2.6462 × 10^2^	2.7651 × 10^2^	1.0770 × 10^2^
F17	mean	2.2757 × 10^3^	1.9957 × 10^3^	1.9544 × 10^3^	2.3000 × 10^3^	2.2977 × 10^3^	2.0751 × 10^3^	1.9801 × 10^3^	2.4452 × 10^3^	2.3502 × 10^3^	2.1687 × 10^3^	2.2254 × 10^3^	1.8574 × 10^3^
	std	2.7626 × 10^2^	1.6987 × 10^2^	1.1393 × 10^2^	1.2199 × 10^2^	2.7606 × 10^2^	2.0310 × 10^2^	9.1128 × 10^1^	2.4003 × 10^2^	2.4509 × 10^2^	2.2021 × 10^2^	1.8528 × 10^2^	2.9121 × 10^1^
F18	mean	4.1652 × 10^5^	6.4123 × 10^5^	1.1114 × 10^5^	4.0719 × 10^6^	4.6263 × 10^4^	6.7045 × 10^5^	5.3089 × 10^3^	1.6027 × 10^4^	9.4520 × 10^4^	6.2307 × 10^4^	1.2719 × 10^5^	2.7823 × 10^3^
	std	3.3795 × 10^5^	5.7206 × 10^5^	8.4463 × 10^4^	1.5024 × 10^6^	3.6244 × 10^4^	4.6140 × 10^5^	4.4383 × 10^3^	1.6863 × 10^4^	9.4900 × 10^4^	3.7934 × 10^4^	6.3967 × 10^4^	3.2598 × 10^2^
F19	mean	1.0421 × 10^4^	1.3416 × 10^4^	5.7798 × 10^4^	4.1086 × 10^3^	4.6849 × 10^3^	1.2598 × 10^4^	2.2757 × 10^3^	7.6820 × 10^3^	6.5206 × 10^5^	9.8353 × 10^3^	7.9636 × 10^5^	2.1558 × 10^3^
	std	1.0218 × 10^4^	1.7150 × 10^4^	6.4123 × 10^4^	2.6330 × 10^3^	2.7085 × 10^3^	1.4065 × 10^4^	3.5961 × 10^2^	8.3638 × 10^3^	2.8344 × 10^6^	9.4090 × 10^3^	7.2657 × 10^5^	7.1828 × 10^1^
F20	mean	2.5924 × 10^3^	2.3682 × 10^3^	2.3296 × 10^3^	2.6797 × 10^3^	2.4606 × 10^3^	2.4191 × 10^3^	2.3063 × 10^3^	2.6804 × 10^3^	2.5009 × 10^3^	2.4945 × 10^3^	2.4323 × 10^3^	2.2592 × 10^3^
	std	1.8659 × 10^2^	1.6589 × 10^2^	1.2553 × 10^2^	1.4716 × 10^2^	1.6846 × 10^2^	1.8311 × 10^2^	6.7487 × 10^1^	1.8067 × 10^2^	1.6437 × 10^2^	1.8614 × 10^2^	1.2110 × 10^2^	3.5669 × 10^1^
F21	mean	2.4777 × 10^3^	2.3837 × 10^3^	2.3806 × 10^3^	2.5069 × 10^3^	2.4340 × 10^3^	2.3818 × 10^3^	2.4849 × 10^3^	2.4545 × 10^3^	2.5256 × 10^3^	2.4444 × 10^3^	2.5237 × 10^3^	2.3651 × 10^3^
	std	3.0931 × 10^1^	3.8093 × 10^1^	2.0688 × 10^1^	1.4038 × 10^1^	5.5433 × 10^1^	2.2668 × 10^1^	5.1195 × 10^1^	3.7596 × 10^1^	5.1834 × 10^1^	3.1345 × 10^1^	4.0025 × 10^1^	7.7143 × 10^1^
F22	mean	5.1053 × 10^3^	4.4880 × 10^3^	3.1461 × 10^3^	4.0745 × 10^3^	2.4836 × 10^3^	4.2215 × 10^3^	5.2023 × 10^3^	4.4967 × 10^3^	5.7815 × 10^3^	2.9570 × 10^3^	4.4516 × 10^3^	2.3765 × 10^3^
	std	2.0751 × 10^3^	2.6396 × 10^3^	1.6627 × 10^3^	1.9327 × 10^3^	9.9718 × 10^2^	1.9367 × 10^3^	1.0728 × 10^3^	2.2931 × 10^3^	1.8194 × 10^3^	1.5182 × 10^3^	1.4247 × 10^3^	2.4947 × 10^1^
F23	mean	3.1018 × 10^3^	2.7224 × 10^3^	2.7458 × 10^3^	2.8600 × 10^3^	2.8650 × 10^3^	2.7452 × 10^3^	2.9335 × 10^3^	2.8736 × 10^3^	3.0395 × 10^3^	2.8418 × 10^3^	2.9698 × 10^3^	2.8011 × 10^3^
	std	1.3152 × 10^2^	2.8661 × 10^1^	2.5012 × 10^1^	8.2729	7.6955 × 10^1^	2.7605 × 10^1^	5.8486 × 10^1^	5.3752 × 10^1^	9.9087 × 10^1^	5.4453 × 10^1^	7.7357 × 10^1^	2.2629 × 10^1^
F24	mean	3.1921 × 10^3^	2.9489 × 10^3^	2.9055 × 10^3^	3.0254 × 10^3^	3.0159 × 10^3^	2.9173 × 10^3^	3.0897 × 10^3^	3.0311 × 10^3^	3.2070 × 10^3^	3.0027 × 10^3^	3.1525 × 10^3^	2.9929 × 10^3^
	std	8.0100 × 10^1^	3.6690 × 10^1^	1.6597 × 10^1^	9.3849	7.1895 × 10^1^	2.2647 × 10^1^	6.9183 × 10^1^	7.0177 × 10^1^	1.1067 × 10^2^	7.1574 × 10^1^	7.9576 × 10^1^	2.3288 × 10^1^
F25	mean	2.8862 × 10^3^	2.8941 × 10^3^	2.9443 × 10^3^	2.8894 × 10^3^	2.8982 × 10^3^	2.8989 × 10^3^	3.5185 × 10^3^	2.8981 × 10^3^	3.0669 × 10^3^	2.8985 × 10^3^	3.2020 × 10^3^	2.9112 × 10^3^
	std	1.5937 × 10^1^	7.3351	2.4522 × 10^1^	1.2179	1.8471 × 10^1^	1.7132 × 10^1^	1.7983 × 10^2^	1.8211 × 10^1^	3.1970 × 10^2^	1.5679 × 10^1^	1.1255 × 10^2^	7.4461
F26	mean	5.2287 × 10^3^	4.1963 × 10^3^	4.6871 × 10^3^	5.7601 × 10^3^	5.2996 × 10^3^	4.6270 × 10^3^	6.9391 × 10^3^	5.7909 × 10^3^	7.6339 × 10^3^	4.9279 × 10^3^	6.7626 × 10^3^	3.3227 × 10^3^
	std	2.1491 × 10^3^	3.2743 × 10^2^	8.2109 × 10^2^	9.9905 × 10^1^	1.6888 × 10^3^	4.5536 × 10^2^	1.2496 × 10^3^	1.1924 × 10^3^	1.6083 × 10^3^	1.4457 × 10^3^	1.5083 × 10^3^	1.2958 × 10^2^
F27	mean	3.2838 × 10^3^	3.2188 × 10^3^	3.2598 × 10^3^	3.2236 × 10^3^	3.3012 × 10^3^	3.2299 × 10^3^	3.3003 × 10^3^	3.2527 × 10^3^	3.3877 × 10^3^	3.2570 × 10^3^	3.3383 × 10^3^	3.2000 × 10^3^
	std	1.5555 × 10^2^	7.2278	2.1788 × 10^1^	5.1804	5.9822 × 10^1^	1.4900 × 10^1^	5.0375 × 10^1^	3.1642 × 10^1^	1.1881 × 10^2^	2.6007 × 10^1^	6.3667 × 10^1^	4.2898 × 10^−5^
F28	mean	3.2236 × 10^3^	3.2622 × 10^3^	3.2915 × 10^3^	3.2725 × 10^3^	3.2191 × 10^3^	3.2652 × 10^3^	4.4828 × 10^3^	3.1547 × 10^3^	3.5331 × 10^3^	3.2141 × 10^3^	3.6918 × 10^3^	3.2425 × 10^3^
	std	2.3672 × 10^1^	2.0194 × 10^1^	1.9313 × 10^1^	1.6807 × 10^1^	2.2995 × 10^1^	2.4976 × 10^1^	4.9612 × 10^2^	6.0590 × 10^1^	6.8972 × 10^2^	1.6065 × 10^1^	2.6725 × 10^2^	1.6663 × 10^1^
F29	mean	4.0703 × 10^3^	3.7420 × 10^3^	3.8102 × 10^3^	4.2967 × 10^3^	4.1527 × 10^3^	3.8297 × 10^3^	4.1396 × 10^3^	4.0470 × 10^3^	4.6176 × 10^3^	4.0132 × 10^3^	4.3910 × 10^3^	3.7283 × 10^3^
	std	2.2803 × 10^2^	1.6776 × 10^2^	1.8370 × 10^2^	1.6946 × 10^2^	3.6060 × 10^2^	1.8165 × 10^2^	2.2584 × 10^2^	2.5266 × 10^2^	5.5424 × 10^2^	2.6034 × 10^2^	3.6668 × 10^2^	9.7067 × 10^1^
F30	mean	4.6868 × 10^4^	2.8949 × 10^4^	1.3896 × 10^6^	1.6655 × 10^5^	1.0816 × 10^4^	1.8075 × 10^5^	8.5158 × 10^4^	9.1788 × 10^3^	1.1683 × 10^6^	1.7620 × 10^4^	6.9311 × 10^6^	3.2936 × 10^3^
	std	3.3144 × 10^4^	2.7233 × 10^4^	8.8381 × 10^5^	1.6426 × 10^5^	3.8082 × 10^3^	1.7661 × 10^5^	9.8783 × 10^4^	2.6490 × 10^3^	9.7566 × 10^5^	8.4497 × 10^3^	3.9271 × 10^6^	2.5829 × 10^1^

**Table A4:** Results of various algorithms tested on the CEC 2017 benchmark (dim = 50).

ID	Metric	PSO	SO	CSA	DMO	GTO	RIME	IAO	RTH	BKA	GKSO	POA	MIPOA
F1	mean	8.5322 × 10^6^	1.3182 × 10^8^	8.1797 × 10^7^	7.1299 × 10^8^	2.6675 × 10^4^	4.5746 × 10^6^	7.1322 × 10^10^	4.0937 × 10^3^	3.0161 × 10^10^	5.2067 × 10^3^	3.8340 × 10^10^	4.3821 × 10^9^
	std	3.6061 × 10^6^	5.8847 × 10^7^	3.4855 × 10^7^	1.6615 × 10^8^	7.0218 × 10^4^	1.5657 × 10^6^	1.2040 × 10^10^	5.4955 × 10^3^	3.3311 × 10^10^	4.1093 × 10^3^	1.0412 × 10^10^	1.3130 × 10^9^
F2	mean	3.3451 × 10^23^	1.0677 × 10^50^	7.0058 × 10^48^	8.0154 × 10^65^	1.3052 × 10^51^	3.7055 × 10^31^	1.9450 × 10^65^	1.9841 × 10^38^	2.7610 × 10^73^	1.5453 × 10^33^	2.7359 × 10^62^	1.1111 × 10^39^
	std	1.1709 × 10^24^	3.9862 × 10^50^	3.5959 × 10^49^	3.3513 × 10^66^	7.1481 × 10^51^	1.8617 × 10^32^	6.6565 × 10^65^	7.7519 × 10^38^	1.3339 × 10^74^	8.1890 × 10^33^	1.4980 × 10^63^	1.1899 × 10^39^
F3	mean	7.4690 × 10^4^	1.3921 × 10^5^	1.0563 × 10^5^	2.8142 × 10^5^	2.6173 × 10^4^	8.0602 × 10^4^	1.0691 × 10^5^	5.8927 × 10^2^	5.6031 × 10^4^	1.1737 × 10^4^	8.8027 × 10^4^	2.1100 × 10^4^
	std	1.8443 × 10^4^	1.7109 × 10^4^	1.4670 × 10^4^	3.0320 × 10^4^	9.8587 × 10^3^	2.0445 × 10^4^	1.6013 × 10^4^	3.3787 × 10^2^	4.0457 × 10^4^	3.7402 × 10^3^	1.6103 × 10^4^	1.9769 × 10^3^
F4	mean	5.4667 × 10^2^	6.3422 × 10^2^	7.4275 × 10^2^	8.7299 × 10^2^	5.6879 × 10^2^	6.3063 × 10^2^	1.7383 × 10^4^	4.6621 × 10^2^	4.5076 × 10^3^	5.8937 × 10^2^	6.9702 × 10^3^	8.2683 × 10^2^
	std	4.9719 × 10^1^	1.2174 × 10^1^	6.9804 × 10^1^	5.8284 × 10^1^	5.9510 × 10^1^	4.6641 × 10^1^	4.2194 × 10^3^	4.4528 × 10^1^	7.2882 × 10^3^	6.2342 × 10^1^	2.8651 × 10^3^	6.8704 × 10^1^
F5	mean	7.7232 × 10^2^	6.4464 × 10^2^	7.1702 × 10^2^	9.6773 × 10^2^	8.3540 × 10^2^	6.8552 × 10^2^	1.0094 × 10^3^	8.2336 × 10^2^	8.9177 × 10^2^	8.2522 × 10^2^	9.1650 × 10^2^	8.1200 × 10^2^
	std	4.2135 × 10^1^	4.9101 × 10^1^	3.2432 × 10^1^	1.7980 × 10^1^	3.8732 × 10^1^	3.9257 × 10^1^	2.9639 × 10^1^	3.7643 × 10^1^	1.0364 × 10^2^	3.7116 × 10^1^	4.0602 × 10^1^	2.2220 × 10^1^
F6	mean	6.5314 × 10^2^	6.0461 × 10^2^	6.3610 × 10^2^	6.1368 × 10^2^	6.5278 × 10^2^	6.1467 × 10^2^	6.6799 × 10^2^	6.4975 × 10^2^	6.6623 × 10^2^	6.5151 × 10^2^	6.6895 × 10^2^	6.5396 × 10^2^
	std	4.8792	9.9519 × 10^−1^	4.6832	1.4701	8.4103	4.6619	7.8117	6.2674	6.0349	5.8286	5.7334	3.2350
F7	mean	1.0872 × 10^3^	8.8242 × 10^2^	1.2364 × 10^3^	1.2949 × 10^3^	1.4708 × 10^3^	9.8869 × 10^2^	1.6877 × 10^3^	1.4670 × 10^3^	1.6924 × 10^3^	1.2569 × 10^3^	1.7833 × 10^3^	1.3906 × 10^3^
	std	5.6970 × 10^1^	2.6896 × 10^1^	8.4577 × 10^1^	2.7303 × 10^1^	1.4863 × 10^2^	3.8671 × 10^1^	1.0677 × 10^2^	1.2227 × 10^2^	1.1478 × 10^2^	1.0757 × 10^2^	5.8390 × 10^1^	6.8404 × 10^1^
F8	mean	1.0936 × 10^3^	9.6922 × 10^2^	9.9360 × 10^2^	1.2712 × 10^3^	1.1297 × 10^3^	1.0037 × 10^3^	1.3071 × 10^3^	1.1298 × 10^3^	1.2083 × 10^3^	1.1214 × 10^3^	1.2415 × 10^3^	1.1279 × 10^3^
	std	3.8918 × 10^1^	7.3059 × 10^1^	4.2196 × 10^1^	2.1085 × 10^1^	5.4943 × 10^1^	5.6722 × 10^1^	4.3024 × 10^1^	4.4701 × 10^1^	8.3692 × 10^1^	3.6631 × 10^1^	2.8555 × 10^1^	1.9082 × 10^1^
F9	mean	2.0387 × 10^4^	1.1524 × 10^3^	5.1556 × 10^3^	6.3521 × 10^3^	1.1516 × 10^4^	5.4498 × 10^3^	2.0472 × 10^4^	1.1213 × 10^4^	1.6058 × 10^4^	9.3874 × 10^3^	1.6463 × 10^4^	1.0997 × 10^4^
	std	4.5273 × 10^3^	1.0518 × 10^2^	1.0245 × 10^3^	1.1536 × 10^3^	1.5536 × 10^3^	3.1976 × 10^3^	2.2451 × 10^3^	1.2094 × 10^3^	5.2639 × 10^3^	2.0255 × 10^3^	1.5556 × 10^3^	9.1815 × 10^2^
F10	mean	7.3083 × 10^3^	1.3086 × 10^4^	8.3853 × 10^3^	1.4984 × 10^4^	9.0466 × 10^3^	7.0884 × 10^3^	1.0457 × 10^4^	7.8641 × 10^3^	9.3145 × 10^3^	8.0249 × 10^3^	8.6790 × 10^3^	6.8332 × 10^3^
	std	8.5951 × 10^2^	1.4486 × 10^3^	1.5879 × 10^3^	3.2774 × 10^2^	1.7814 × 10^3^	7.8758 × 10^2^	5.9894 × 10^2^	8.6321 × 10^2^	2.1231 × 10^3^	8.2220 × 10^2^	9.3685 × 10^2^	4.0617 × 10^2^
F11	mean	1.3210 × 10^3^	1.6238 × 10^3^	2.2796 × 10^3^	3.2735 × 10^3^	1.3240 × 10^3^	1.5604 × 10^3^	8.9185 × 10^3^	1.3396 × 10^3^	3.9391 × 10^3^	1.3115 × 10^3^	5.5198 × 10^3^	1.4967 × 10^3^
	std	6.3277 × 10^1^	1.1931 × 10^2^	3.4834 × 10^2^	4.8088 × 10^2^	4.9314 × 10^1^	1.1055 × 10^2^	3.4274 × 10^3^	7.2954 × 10^1^	5.5897 × 10^3^	3.5898 × 10^1^	1.9716 × 10^3^	4.4170 × 10^1^
F12	mean	1.3110 × 10^7^	1.5498 × 10^7^	1.8457 × 10^8^	3.5719 × 10^8^	3.6492 × 10^6^	8.3871 × 10^7^	1.7431 × 10^10^	2.2180 × 10^5^	3.9297 × 10^9^	3.9625 × 10^6^	9.3462 × 10^9^	4.8477 × 10^7^
	std	7.0417 × 10^6^	1.4563 × 10^7^	9.9101 × 10^7^	1.0246 × 10^8^	2.0557 × 10^6^	6.4504 × 10^7^	8.5258 × 10^9^	1.2964 × 10^5^	1.1955 × 10^10^	3.5687 × 10^6^	7.1303 × 10^9^	1.7245 × 10^7^
F13	mean	2.7805 × 10^4^	5.9732 × 10^4^	1.0392 × 10^5^	5.5730 × 10^3^	1.3001 × 10^4^	1.9913 × 10^5^	1.3113 × 10^9^	9.2662 × 10^3^	5.4103 × 10^8^	1.4522 × 10^4^	2.4145 × 10^9^	1.5360 × 10^5^
	std	1.1439 × 10^4^	5.4680 × 10^4^	7.0988 × 10^4^	1.9041 × 10^3^	8.7837 × 10^3^	9.6190 × 10^4^	2.0765 × 10^9^	8.6996 × 10^3^	1.8483 × 10^9^	8.8842 × 10^3^	3.6263 × 10^9^	4.7933 × 10^4^
F14	mean	9.6615 × 10^4^	1.4835 × 10^5^	1.2913 × 10^5^	1.4499 × 10^6^	2.8051 × 10^4^	2.4830 × 10^5^	1.8883 × 10^3^	4.5322 × 10^3^	1.8367 × 10^5^	2.6122 × 10^4^	2.5267 × 10^5^	1.8003 × 10^3^
	std	7.2069 × 10^4^	9.6998 × 10^4^	1.0661 × 10^5^	5.1251 × 10^5^	2.5840 × 10^4^	1.3932 × 10^5^	1.0230 × 10^2^	1.8902 × 10^3^	3.4754 × 10^5^	2.1630 × 10^4^	1.7517 × 10^5^	4.2632 × 10^1^
F15	mean	1.2254 × 10^4^	2.7373 × 10^4^	3.5084 × 10^4^	1.1215 × 10^4^	1.5084 × 10^4^	5.7688 × 10^4^	2.3708 × 10^4^	9.5876 × 10^3^	9.8547 × 10^7^	8.6000 × 10^3^	5.3648 × 10^7^	1.3201 × 10^4^
	std	1.1324 × 10^4^	2.8152 × 10^4^	2.8007 × 10^4^	4.7279 × 10^3^	9.5968 × 10^3^	2.3589 × 10^4^	1.5003 × 10^4^	8.0147 × 10^3^	3.7479 × 10^8^	6.8860 × 10^3^	1.7192 × 10^8^	3.2327 × 10^3^
F16	mean	3.3719 × 10^3^	3.8673 × 10^3^	3.2003 × 10^3^	5.0709 × 10^3^	3.6610 × 10^3^	3.5224 × 10^3^	4.2102 × 10^3^	3.6202 × 10^3^	3.9494 × 10^3^	3.5683 × 10^3^	4.0847 × 10^3^	2.8881 × 10^3^
	std	3.9454 × 10^2^	6.1406 × 10^2^	3.9749 × 10^2^	2.0937 × 10^2^	3.5012 × 10^2^	3.6666 × 10^2^	4.3956 × 10^2^	3.7989 × 10^2^	7.6131 × 10^2^	3.8583 × 10^2^	7.0843 × 10^2^	1.4197 × 10^2^
F17	mean	3.0972 × 10^3^	3.2048 × 10^3^	2.9675 × 10^3^	3.9518 × 10^3^	3.3964 × 10^3^	3.2739 × 10^3^	3.0417 × 10^3^	3.5562 × 10^3^	3.3776 × 10^3^	3.2090 × 10^3^	3.4619 × 10^3^	2.6771 × 10^3^
	std	2.6084 × 10^2^	4.4289 × 10^2^	2.5697 × 10^2^	1.8102 × 10^2^	3.0592 × 10^2^	3.9052 × 10^2^	3.1113 × 10^2^	3.7638 × 10^2^	2.4464 × 10^2^	4.1170 × 10^2^	4.2054 × 10^2^	1.0304 × 10^2^
F18	mean	1.9105 × 10^6^	3.3631 × 10^6^	9.0861 × 10^5^	2.0171 × 10^7^	2.1176 × 10^5^	3.8774 × 10^6^	8.0538 × 10^4^	4.6191 × 10^4^	1.6569 × 10^6^	1.9241 × 10^5^	2.1752 × 10^6^	1.4743 × 10^4^
	std	1.1776 × 10^6^	2.8940 × 10^6^	6.4474 × 10^5^	7.3470 × 10^6^	1.5122 × 10^5^	2.7825 × 10^6^	3.8083 × 10^4^	2.8165 × 10^4^	6.2137 × 10^6^	1.1711 × 10^5^	1.6950 × 10^6^	4.3043 × 10^3^
F19	mean	1.4261 × 10^4^	2.0841 × 10^4^	1.1195 × 10^6^	1.5620 × 10^4^	1.6993 × 10^4^	7.0230 × 10^4^	1.5301 × 10^5^	1.7019 × 10^4^	2.9209 × 10^7^	1.9359 × 10^4^	6.9796 × 10^6^	4.0188 × 10^4^
	std	1.0451 × 10^4^	1.2092 × 10^4^	1.6224 × 10^6^	5.8890 × 10^3^	1.0320 × 10^4^	4.2397 × 10^4^	2.0527 × 10^5^	1.2338 × 10^4^	8.8353 × 10^7^	1.2147 × 10^4^	9.0392 × 10^6^	1.7572 × 10^4^
F20	mean	3.1793 × 10^3^	3.3837 × 10^3^	2.8044 × 10^3^	4.0246 × 10^3^	3.1375 × 10^3^	3.0549 × 10^3^	2.8223 × 10^3^	3.3734 × 10^3^	3.1326 × 10^3^	3.1334 × 10^3^	3.0664 × 10^3^	2.5980 × 10^3^
	std	3.0554 × 10^2^	3.6004 × 10^2^	2.9719 × 10^2^	1.6373 × 10^2^	3.1284 × 10^2^	3.1447 × 10^2^	1.6460 × 10^2^	3.4478 × 10^2^	1.9898 × 10^2^	2.8808 × 10^2^	2.0241 × 10^2^	7.4337 × 10^1^
F21	mean	2.6740 × 10^3^	2.4860 × 10^3^	2.4984 × 10^3^	2.7636 × 10^3^	2.6113 × 10^3^	2.4862 × 10^3^	2.8268 × 10^3^	2.6569 × 10^3^	2.8345 × 10^3^	2.6105 × 10^3^	2.7526 × 10^3^	2.5992 × 10^3^
	std	7.1743 × 10^1^	6.7934 × 10^1^	3.7843 × 10^1^	1.7255 × 10^1^	5.6371 × 10^1^	4.5008 × 10^1^	5.9263 × 10^1^	8.5106 × 10^1^	1.2556 × 10^2^	7.0332 × 10^1^	5.8617 × 10^1^	1.9368 × 10^1^
F22	mean	9.5696 × 10^3^	1.4445 × 10^4^	1.0385 × 10^4^	1.6330 × 10^4^	1.0240 × 10^4^	9.0811 × 10^3^	1.2799 × 10^4^	9.4962 × 10^3^	1.0677 × 10^4^	9.5255 × 10^3^	1.1190 × 10^4^	7.4436 × 10^3^
	std	8.0283 × 10^2^	1.5477 × 10^3^	1.5686 × 10^3^	3.9679 × 10^2^	2.5635 × 10^3^	8.7517 × 10^2^	6.0063 × 10^2^	8.3193 × 10^2^	1.6694 × 10^3^	9.7338 × 10^2^	1.3694 × 10^3^	2.0235 × 10^3^
F23	mean	3.5653 × 10^3^	2.8703 × 10^3^	3.0335 × 10^3^	3.1867 × 10^3^	3.2445 × 10^3^	2.9644 × 10^3^	3.5057 × 10^3^	3.2067 × 10^3^	3.6776 × 10^3^	3.1718 × 10^3^	3.4901 × 10^3^	3.1545 × 10^3^
	std	1.6994 × 10^2^	3.3192 × 10^1^	6.8988 × 10^1^	1.7828 × 10^1^	1.0191 × 10^2^	4.6745 × 10^1^	1.2350 × 10^2^	1.2165 × 10^2^	1.5194 × 10^2^	8.3427 × 10^1^	1.2886 × 10^2^	3.4892 × 10^1^
F24	mean	3.5423 × 10^3^	3.2021 × 10^3^	3.1310 × 10^3^	3.3266 × 10^3^	3.3340 × 10^3^	3.1255 × 10^3^	3.5924 × 10^3^	3.3745 × 10^3^	3.7701 × 10^3^	3.3671 × 10^3^	3.6480 × 10^3^	3.3511 × 10^3^
	std	1.2730 × 10^2^	7.4338 × 10^1^	5.1960 × 10^1^	1.4939 × 10^1^	1.0669 × 10^2^	6.6194 × 10^1^	1.2112 × 10^2^	1.1087 × 10^2^	1.2268 × 10^2^	1.1360 × 10^2^	1.0341 × 10^2^	4.9728 × 10^1^
F25	mean	2.9978 × 10^3^	3.0793 × 10^3^	3.2971 × 10^3^	3.3158 × 10^3^	3.0976 × 10^3^	3.0915 × 10^3^	1.0230 × 10^4^	3.0514 × 10^3^	4.4812 × 10^3^	3.0738 × 10^3^	5.8084 × 10^3^	3.3121 × 10^3^
	std	4.9334 × 10^1^	1.5929 × 10^1^	6.9890 × 10^1^	5.3084 × 10^1^	3.7034 × 10^1^	3.7980 × 10^1^	1.3500 × 10^3^	3.4700 × 10^1^	2.0357 × 10^3^	2.7891 × 10^1^	1.0673 × 10^3^	5.7403 × 10^1^
F26	mean	8.5450 × 10^3^	5.0509 × 10^3^	7.6002 × 10^3^	8.3700 × 10^3^	7.8072 × 10^3^	6.0831 × 10^3^	1.3754 × 10^4^	9.0507 × 10^3^	1.1233 × 10^4^	7.3313 × 10^3^	1.2268 × 10^4^	5.5019 × 10^3^
	std	3.2402 × 10^3^	3.3582 × 10^2^	1.1500 × 10^3^	1.6438 × 10^2^	2.7339 × 10^3^	6.1392 × 10^2^	1.1604 × 10^3^	2.4138 × 10^3^	2.2651 × 10^3^	3.6809 × 10^3^	1.7113 × 10^3^	6.1053 × 10^2^
F27	mean	4.0427 × 10^3^	3.3896 × 10^3^	3.7181 × 10^3^	3.5280 × 10^3^	3.8386 × 10^3^	3.5050 × 10^3^	3.8432 × 10^3^	3.5780 × 10^3^	4.0038 × 10^3^	3.6703 × 10^3^	4.0771 × 10^3^	3.2000 × 10^3^
	std	6.3349 × 10^2^	4.3684 × 10^1^	1.4754 × 10^2^	3.0012 × 10^1^	3.4820 × 10^2^	1.0518 × 10^2^	1.8209 × 10^2^	1.4643 × 10^2^	3.1717 × 10^2^	1.4539 × 10^2^	2.4562 × 10^2^	5.5029 × 10^−5^
F28	mean	3.2966 × 10^3^	3.3696 × 10^3^	3.7508 × 10^3^	3.8183 × 10^3^	3.3694 × 10^3^	3.3640 × 10^3^	8.8216 × 10^3^	3.3001 × 10^3^	4.6452 × 10^3^	3.3275 × 10^3^	6.1658 × 10^3^	3.3000 × 10^3^
	std	3.2128 × 10^1^	2.5421 × 10^1^	1.1214 × 10^2^	1.2820 × 10^2^	4.7126 × 10^1^	2.9154 × 10^1^	1.0316 × 10^3^	3.6259 × 10^1^	1.5556 × 10^3^	2.6159 × 10^1^	6.5590 × 10^2^	1.2179 × 10^−4^
F29	mean	5.1317 × 10^3^	4.2208 × 10^3^	5.1651 × 10^3^	5.6879 × 10^3^	5.1170 × 10^3^	4.7550 × 10^3^	6.7152 × 10^3^	4.8689 × 10^3^	7.4650 × 10^3^	5.1252 × 10^3^	6.6963 × 10^3^	4.7386 × 10^3^
	std	2.6153 × 10^2^	3.7565 × 10^2^	4.7302 × 10^2^	2.3821 × 10^2^	3.8304 × 10^2^	4.2509 × 10^2^	1.1421 × 10^3^	5.2737 × 10^2^	3.1215 × 10^3^	3.4228 × 10^2^	8.7449 × 10^2^	3.2062 × 10^2^
F30	mean	4.8818 × 10^6^	2.0997 × 10^6^	9.8432 × 10^7^	1.7188 × 10^7^	1.2291 × 10^6^	2.2362 × 10^7^	5.0711 × 10^7^	8.4395 × 10^5^	6.9653 × 10^7^	5.5415 × 10^6^	1.7851 × 10^8^	4.5999 × 10^3^
	std	2.1182 × 10^6^	7.3139 × 10^5^	4.0720 × 10^7^	8.5103 × 10^6^	3.2638 × 10^5^	7.7087 × 10^6^	4.9564 × 10^7^	1.6501 × 10^5^	1.2096 × 10^8^	2.3665 × 10^6^	8.7983 × 10^7^	7.2432 × 10^2^

**Table A5:** *p*-values for various algorithms on the CEC 2017 (dim = 10).

Item	PSO	SO	CSA	DMO	GTO	RIME	IAO	RTH	BKA	GKSO	POA
F1	3.6897 × 10^−11^	1.2057 × 10^−10^	2.6099 × 10^−10^	3.0199 × 10^−11^	5.0723 × 10^−10^	3.0199 × 10^−11^	4.0988 × 10^−12^	3.5012 × 10^−3^	3.0199 × 10^−11^	3.0199 × 10^−11^	3.0199 × 10^−11^
F2	3.0199 × 10^−11^	1.6810 × 10^−8^	1.6572 × 10^−11^	6.2258 × 10^−10^	5.0628 × 10^−6^	2.9329 × 10^−5^	2.1419 × 10^−2^	4.1926 × 10^−2^	1.9381 × 10^−9^	3.3371 × 10^−1^	5.7678 × 10^−11^
F3	1.2057 × 10^−10^	8.1014 × 10^−10^	7.1021 × 10^−10^	3.0199 × 10^−11^	2.8422 × 10^−11^	3.0199 × 10^−11^	3.1507 × 10^−12^	1.1364 × 10^−11^	3.0199 × 10^−11^	3.2686 × 10^−11^	3.0199 × 10^−11^
F4	2.6099 × 10^−10^	3.0199 × 10^−11^	3.0199 × 10^−11^	3.0199 × 10^−11^	4.6427 × 10^−1^	3.0199 × 10^−11^	2.1822 × 10^−11^	2.7652 × 10^−11^	1.4643 × 10^−10^	3.8710 × 10^−1^	3.0199 × 10^−11^
F5	3.0199 × 10^−11^	9.0490 × 10^−2^	7.4774 × 10^−2^	7.3891 × 10^−11^	2.5682 × 10^−7^	6.6273 × 10^−1^	6.3533 × 10^−2^	7.3846 × 10^−11^	5.4617 × 10^−9^	7.0430 × 10^−7^	3.0199 × 10^−11^
F6	3.0811 × 10^−8^	3.4742 × 10^−10^	3.0103 × 10^−7^	1.2118 × 10^−12^	1.8567 × 10^−9^	9.9186 × 10^−11^	5.5699 × 10^−3^	6.7220 × 10^−10^	3.0199 × 10^−11^	1.2597 × 10^−1^	3.0199 × 10^−11^
F7	3.6439 × 10^−2^	6.1452 × 10^−2^	5.0114 × 10^−1^	3.4742 × 10^−10^	2.3715 × 10^−10^	6.3088 × 10^−1^	9.0000 × 10^−1^	3.3384 × 10^−11^	3.0199 × 10^−11^	2.7829 × 10^−7^	3.0199 × 10^−11^
F8	5.8587 × 10^−6^	7.2446 × 10^−2^	3.0001 × 10^−4^	3.1589 × 10^−10^	3.3520 × 10^−8^	2.3800 × 10^−3^	6.5204 × 10^−1^	7.3683 × 10^−10^	5.0912 × 10^−6^	1.8602 × 10^−6^	3.0199 × 10^−11^
F9	1.0666 × 10^−7^	8.9999 × 10^−1^	3.1589 × 10^−10^	1.2118 × 10^−12^	1.0648 × 10^−7^	8.6844 × 10^−3^	4.6120 × 10^−5^	5.5727 × 10^−10^	3.0199 × 10^−11^	1.0892 × 10^−5^	3.0199 × 10^−11^
F10	3.0199 × 10^−11^	6.7869 × 10^−2^	2.0275 × 10^−7^	3.0199 × 10^−11^	3.0199 × 10^−11^	6.3560 × 10^−5^	1.8731 × 10^−7^	9.9186 × 10^−11^	3.0199 × 10^−11^	2.4386 × 10^−9^	6.0104 × 10^−8^
F11	3.0199 × 10^−11^	7.2446 × 10^−2^	1.1737 × 10^−9^	9.6263 × 10^−2^	7.5991 × 10^−7^	5.1857 × 10^−7^	1.1674 × 10^−5^	1.0702 × 10^−9^	1.6947 × 10^−9^	3.8307 × 10^−5^	4.9752 × 10^−11^
F12	3.0199 × 10^−11^	3.0199 × 10^−11^	3.0199 × 10^−11^	3.0199 × 10^−11^	3.0199 × 10^−11^	3.0199 × 10^−11^	1.7142 × 10^−1^	3.0199 × 10^−11^	3.0199 × 10^−11^	3.0199 × 10^−11^	3.0199 × 10^−11^
F13	3.0199 × 10^−11^	3.0199 × 10^−11^	3.0199 × 10^−11^	3.0199 × 10^−11^	1.9568 × 10^−10^	3.0199 × 10^−11^	1.4932 × 10^−4^	1.3289 × 10^−10^	3.0199 × 10^−11^	3.0199 × 10^−11^	3.0199 × 10^−11^
F14	3.0199 × 10^−11^	3.0199 × 10^−11^	3.0199 × 10^−11^	3.0199 × 10^−11^	1.9568 × 10^−10^	8.8910 × 10^−10^	6.5261 × 10^−7^	3.0199 × 10^−11^	3.0199 × 10^−11^	5.9673 × 10^−9^	3.0199 × 10^−11^
F15	3.0199 × 10^−11^	3.0199 × 10^−11^	3.0199 × 10^−11^	3.0199 × 10^−11^	4.5726 × 10^−9^	3.0199 × 10^−11^	4.0772 × 10^−11^	3.0199 × 10^−11^	3.0199 × 10^−11^	3.4971 × 10^−9^	3.0199 × 10^−11^
F16	3.1967 × 10^−9^	8.5000 × 10^−2^	5.9673 × 10^−9^	2.8716 × 10^−10^	3.4971 × 10^−9^	2.4913 × 10^−6^	2.9205 × 10^−2^	2.6695 × 10^−9^	4.1997 × 10^−10^	5.9706 × 10^−5^	3.0199 × 10^−11^
F17	3.5201 × 10^−7^	4.4642 × 10^−1^	7.1988 × 10^−5^	3.5708 × 10^−6^	5.6073 × 10^−5^	2.2823 × 10^−1^	5.0120 × 10^−2^	1.6980 × 10^−8^	5.0723 × 10^−10^	2.4157 × 10^−2^	8.9934 × 10^−11^
F18	3.0199 × 10^−11^	3.0199 × 10^−11^	3.0199 × 10^−11^	3.0199 × 10^−11^	2.3715 × 10^−10^	3.0199 × 10^−11^	4.0330 × 10^−3^	4.5043 × 10^−11^	3.0199 × 10^−11^	3.0199 × 10^−11^	3.0199 × 10^−11^
F19	3.0199 × 10^−11^	3.0199 × 10^−11^	3.0199 × 10^−11^	3.0199 × 10^−11^	3.0199 × 10^−11^	1.4110 × 10^−9^	9.0632 × 10^−8^	3.0199 × 10^−11^	3.0199 × 10^−11^	3.0199 × 10^−11^	3.0199 × 10^−11^
F20	2.6099 × 10^−10^	1.2235 × 10^−1^	5.2650 × 10^−5^	3.0199 × 10^−11^	1.1023 × 10^−8^	1.4932 × 10^−4^	7.2827 × 10^−1^	3.0199 × 10^−11^	1.0702 × 10^−9^	6.6689 × 10^−3^	2.2273 × 10^−9^
F21	2.9590 × 10^−5^	3.0199 × 10^−11^	7.1152 × 10^−9^	3.0199 × 10^−11^	5.3672 × 10^−2^	3.0199 × 10^−11^	1.0276 × 10^−7^	5.0723 × 10^−10^	3.0199 × 10^−11^	1.2988 × 10^−3^	3.0199 × 10^−11^
F22	1.4110 × 10^−9^	9.9186 × 10^−11^	8.8411 × 10^−7^	1.8577 × 10^−1^	7.3803 × 10^−10^	5.5999 × 10^−7^	2.4155 × 10^−2^	2.0338 × 10^−9^	3.0199 × 10^−11^	7.1186 × 10^−9^	3.8249 × 10^−9^
F23	3.0199 × 10^−11^	2.0621 × 10^−1^	3.0317 × 10^−2^	1.6980 × 10^−8^	2.5721 × 10^−7^	7.1988 × 10^−5^	8.0727 × 10^−1^	6.1210 × 10^−10^	2.4386 × 10^−9^	5.4941 × 10^−11^	8.9934 × 10^−11^
F24	2.1327 × 10^−5^	3.0199 × 10^−11^	4.1997 × 10^−10^	3.0199 × 10^−11^	2.1305 × 10^−5^	3.0199 × 10^−11^	3.3595 × 10^−2^	4.1997 × 10^−10^	3.0199 × 10^−11^	5.5591 × 10^−4^	3.0199 × 10^−11^
F25	3.2555 × 10^−7^	5.5727 × 10^−10^	3.0199 × 10^−11^	3.0199 × 10^−11^	5.0723 × 10^−10^	3.0199 × 10^−11^	2.7500 × 10^−3^	5.5727 × 10^−10^	3.0103 × 10^−7^	2.8700 × 10^−10^	3.0199 × 10^−11^
F26	1.6947 × 10^−9^	5.4941 × 10^−11^	2.4982 × 10^−11^	8.0555 × 10^−11^	2.9045 × 10^−11^	1.3289 × 10^−10^	1.2422 × 10^−7^	5.4773 × 10^−11^	5.4941 × 10^−11^	1.6755 × 10^−9^	9.9186 × 10^−11^
F27	3.0199 × 10^−11^	3.0199 × 10^−11^	3.0180 × 10^−11^	3.0199 × 10^−11^	3.0180 × 10^−11^	3.0199 × 10^−11^	2.9580 × 10^−11^	3.0199 × 10^−11^	3.0199 × 10^−11^	3.0199 × 10^−11^	3.0199 × 10^−11^
F28	3.5012 × 10^−3^	4.4429 × 10^−10^	1.6168 × 10^−11^	3.0199 × 10^−11^	9.8230 × 10^−1^	3.0199 × 10^−11^	1.4319 × 10^−2^	1.6722 × 10^−4^	3.0199 × 10^−11^	1.2211 × 10^−2^	3.0199 × 10^−11^
F29	3.0199 × 10^−11^	7.0430 × 10^−7^	1.0407 × 10^−4^	3.0199 × 10^−11^	1.1077 × 10^−6^	1.2541 × 10^−7^	7.2827 × 10^−1^	3.0199 × 10^−11^	2.1544 × 10^−10^	2.6695 × 10^−9^	5.4941 × 10^−11^
F30	3.0199 × 10^−11^	3.0199 × 10^−11^	3.0199 × 10^−11^	3.0199 × 10^−11^	3.0142 × 10^−11^	3.0199 × 10^−11^	3.0199 × 10^−11^	3.0142 × 10^−11^	3.0199 × 10^−11^	3.0199 × 10^−11^	3.0199 × 10^−11^

**Table A6:** *p*-values for various algorithms on the CEC 2017 (dim = 30).

Item	PSO	SO	CSA	DMO	GTO	RIME	IAO	RTH	BKA	GKSO	POA
F1	3.0199 × 10^−11^	1.2057 × 10^−10^	3.0199 × 10^−11^	3.0199 × 10^−11^	3.0199 × 10^−11^	3.0199 × 10^−11^	3.0199 × 10^−11^	3.0199 × 10^−11^	2.6695 × 10^−9^	3.0199 × 10^−11^	3.0199 × 10^−11^
F2	3.0199 × 10^−11^	6.1210 × 10^−10^	3.0199 × 10^−11^	3.0199 × 10^−11^	2.6243 × 10^−3^	1.1737 × 10^−9^	3.0199 × 10^−11^	1.6980 × 10^−8^	5.9673 × 10^−9^	1.2057 × 10^−10^	3.0199 × 10^−11^
F3	3.0199 × 10^−11^	3.0199 × 10^−11^	3.0199 × 10^−11^	3.0199 × 10^−11^	1.1738 × 10^−3^	6.0658 × 10^−11^	3.0199 × 10^−11^	3.0199 × 10^−11^	3.0199 × 10^−11^	3.0199 × 10^−11^	3.0199 × 10^−11^
F4	3.3520 × 10^−8^	6.2040 × 10^−1^	3.9167 × 10^−2^	1.2541 × 10^−7^	7.3940 × 10^−1^	2.8913 × 10^−3^	3.0199 × 10^−11^	5.4907 × 10^−11^	4.6856 × 10^−8^	9.0490 × 10^−2^	3.0199 × 10^−11^
F5	2.3800 × 10^−3^	3.0199 × 10^−11^	3.3384 × 10^−11^	3.0199 × 10^−11^	3.5923 × 10^−5^	4.5043 × 10^−11^	6.7220 × 10^−10^	1.7649 × 10^−2^	1.7769 × 10^−10^	2.5188 × 10^−1^	3.0199 × 10^−11^
F6	2.4994 × 10^−3^	3.0199 × 10^−11^	2.1544 × 10^−10^	3.0199 × 10^−11^	8.5641 × 10^−4^	3.0199 × 10^−11^	2.6099 × 10^−10^	6.3533 × 10^−2^	3.0199 × 10^−11^	3.5137 × 10^−2^	3.0199 × 10^−11^
F7	8.1014 × 10^−10^	3.0199 × 10^−11^	2.7726 × 10^−5^	1.7145 × 10^−1^	7.2208 × 10^−6^	8.1527 × 10^−11^	1.0937 × 10^−10^	3.0103 × 10^−7^	3.0199 × 10^−11^	2.0621 × 10^−1^	3.0199 × 10^−11^
F8	3.1830 × 10^−1^	4.2175 × 10^−4^	2.3715 × 10^−10^	3.0199 × 10^−11^	6.5261 × 10^−7^	4.6159 × 10^−10^	3.0199 × 10^−11^	1.8916 × 10^−4^	3.1967 × 10^−9^	1.1747 × 10^−4^	3.0199 × 10^−11^
F9	4.3106 × 10^−8^	3.0199 × 10^−11^	3.8202 × 10^−10^	3.0199 × 10^−11^	2.7726 × 10^−5^	1.1937 × 10^−6^	1.6980 × 10^−8^	1.6947 × 10^−9^	3.0199 × 10^−11^	3.7904 × 10^−1^	3.0199 × 10^−11^
F10	3.0103 × 10^−7^	3.0199 × 10^−11^	3.4742 × 10^−10^	3.0199 × 10^−11^	3.0199 × 10^−11^	7.4827 × 10^−2^	3.0199 × 10^−11^	3.0199 × 10^−11^	2.3715 × 10^−10^	1.2860 × 10^−6^	2.3715 × 10^−10^
F11	1.6955 × 10^−2^	1.6798 × 10^−3^	2.9590 × 10^−5^	3.0199 × 10^−11^	6.6273 × 10^−1^	3.3681 × 10^−5^	3.0199 × 10^−11^	5.2978 × 10^−1^	7.5991 × 10^−7^	8.5000 × 10^−2^	3.0199 × 10^−11^
F12	3.0199 × 10^−11^	3.1589 × 10^−10^	3.0199 × 10^−11^	3.0199 × 10^−11^	8.4848 × 10^−9^	3.0199 × 10^−11^	3.0199 × 10^−11^	9.0307 × 10^−4^	3.0199 × 10^−11^	1.6351 × 10^−5^	3.0199 × 10^−11^
F13	2.1702 × 10^−1^	1.8368 × 10^−2^	3.1589 × 10^−10^	1.7649 × 10^−2^	3.5137 × 10^−2^	1.3594 × 10^−7^	1.0547 × 10^−1^	8.2357 × 10^−2^	9.9186 × 10^−11^	1.6798 × 10^−3^	3.0199 × 10^−11^
F14	3.0199 × 10^−11^	3.0199 × 10^−11^	3.0199 × 10^−11^	3.0199 × 10^−11^	2.1947 × 10^−8^	3.0199 × 10^−11^	8.7710 × 10^−2^	4.1997 × 10^−10^	3.0199 × 10^−11^	3.0199 × 10^−11^	3.0199 × 10^−11^
F15	1.8682 × 10^−5^	2.9215 × 10^−9^	3.0199 × 10^−11^	1.9073 × 10^−1^	5.0842 × 10^−3^	2.1959 × 10^−7^	2.6077 × 10^−2^	1.2493 × 10^−5^	3.0199 × 10^−11^	2.1156 × 10^−1^	3.0199 × 10^−11^
F16	4.1825 × 10^−9^	5.5611 × 10^−4^	1.1747 × 10^−4^	3.0199 × 10^−11^	3.1589 × 10^−10^	1.1737 × 10^−9^	9.8329 × 10^−8^	3.8249 × 10^−9^	3.0199 × 10^−11^	5.4617 × 10^−9^	3.0199 × 10^−11^
F17	3.8202 × 10^−10^	4.6371 × 10^−3^	5.5611 × 10^−4^	3.0199 × 10^−11^	3.4971 × 10^−9^	3.9648 × 10^−8^	1.1077 × 10^−6^	3.0199 × 10^−11^	3.6897 × 10^−11^	4.9980 × 10^−9^	9.9186 × 10^−11^
F18	3.0199 × 10^−11^	3.0199 × 10^−11^	3.0199 × 10^−11^	3.0199 × 10^−11^	3.0199 × 10^−11^	3.0199 × 10^−11^	2.3399 × 10^−1^	5.1857 × 10^−7^	3.0199 × 10^−11^	3.0199 × 10^−11^	3.0199 × 10^−11^
F19	4.6856 × 10^−8^	4.1825 × 10^−9^	3.0199 × 10^−11^	1.3111 × 10^−8^	1.2870 × 10^−9^	4.1997 × 10^−10^	9.1171 × 10^−1^	1.2870 × 10^−9^	3.0199 × 10^−11^	9.9186 × 10^−11^	3.0199 × 10^−11^
F20	9.2603 × 10^−9^	2.8913 × 10^−3^	1.5178 × 10^−3^	3.0199 × 10^−11^	7.0430 × 10^−7^	1.1747 × 10^−4^	2.5306 × 10^−4^	3.0199 × 10^−11^	2.8716 × 10^−10^	5.5329 × 10^−8^	9.9186 × 10^−11^
F21	6.6955 × 10^−11^	1.7613 × 10^−1^	2.5101 × 10^−2^	3.0199 × 10^−11^	1.5292 × 10^−5^	7.0127 × 10^−2^	6.7220 × 10^−10^	3.9648 × 10^−8^	3.0199 × 10^−11^	1.1567 × 10^−7^	3.0199 × 10^−11^
F22	2.7086 × 10^−2^	6.7350 × 10^−1^	1.5014 × 10^−2^	3.6459 × 10^−8^	5.5727 × 10^−10^	6.6273 × 10^−1^	3.0199 × 10^−11^	1.0000	1.2057 × 10^−10^	9.5139 × 10^−6^	3.0199 × 10^−11^
F23	3.0199 × 10^−11^	5.0723 × 10^−10^	2.4386 × 10^−9^	3.0199 × 10^−11^	1.8575 × 10^−3^	8.4848 × 10^−9^	4.5043 × 10^−11^	1.5964 × 10^−7^	3.0199 × 10^−11^	6.6689 × 10^−3^	3.0199 × 10^−11^
F24	3.0199 × 10^−11^	1.1077 × 10^−6^	8.9934 × 10^−11^	2.6099 × 10^−10^	3.1830 × 10^−1^	3.4742 × 10^−10^	5.0723 × 10^−10^	1.0547 × 10^−1^	4.5043 × 10^−11^	8.1875 × 10^−1^	3.0199 × 10^−11^
F25	2.1947 × 10^−8^	1.1023 × 10^−8^	2.8790 × 10^−6^	3.3384 × 10^−11^	5.2650 × 10^−5^	2.2539 × 10^−4^	3.0199 × 10^−11^	7.6588 × 10^−5^	7.3891 × 10^−11^	1.0407 × 10^−4^	3.0199 × 10^−11^
F26	3.7904 × 10^−1^	3.0199 × 10^−11^	1.1077 × 10^−6^	3.0199 × 10^−11^	1.9527 × 10^−3^	5.5727 × 10^−10^	3.0199 × 10^−11^	1.0666 × 10^−7^	4.1997 × 10^−10^	7.9590 × 10^−3^	3.0199 × 10^−11^
F27	1.0000	3.0199 × 10^−11^	3.0199 × 10^−11^	3.0199 × 10^−11^	3.0199 × 10^−11^	5.5727 × 10^−10^	3.0199 × 10^−11^	3.0199 × 10^−11^	3.0199 × 10^−11^	3.0199 × 10^−11^	3.0199 × 10^−11^
F28	1.0035 × 10^−3^	3.5638 × 10^−4^	1.9568 × 10^−10^	3.0811 × 10^−8^	1.0407 × 10^−4^	1.3250 × 10^−4^	3.0199 × 10^−11^	6.9935 × 10^−9^	2.3168 × 10^−6^	1.7294 × 10^−7^	3.0199 × 10^−11^
F29	4.5726 × 10^−9^	6.6273 × 10^−1^	1.1536 × 10^−1^	3.0199 × 10^−11^	6.0459 × 10^−7^	4.0330 × 10^−3^	8.1527 × 10^−11^	8.3520 × 10^−8^	3.0199 × 10^−11^	9.5332 × 10^−7^	3.0199 × 10^−11^
F30	3.0199 × 10^−11^	3.0199 × 10^−11^	3.0199 × 10^−11^	3.0199 × 10^−11^	3.0199 × 10^−11^	3.0199 × 10^−11^	3.0199 × 10^−11^	3.0199 × 10^−11^	3.0199 × 10^−11^	3.0199 × 10^−11^	3.0199 × 10^−11^

**Table A7:** *p*-values for various algorithms on the CEC 2017 (dim = 50).

Item	PSO	SO	CSA	DMO	GTO	RIME	IAO	RTH	BKA	GKSO	POA
F1	3.0199 × 10^−11^	3.0199 × 10^−11^	3.0199 × 10^−11^	3.0199 × 10^−11^	3.0199 × 10^−11^	3.0199 × 10^−11^	3.0199 × 10^−11^	3.0199 × 10^−11^	9.5332 × 10^−7^	3.0199 × 10^−11^	3.0199 × 10^−11^
F2	3.0199 × 10^−11^	3.0199 × 10^−11^	1.1737 × 10^−9^	3.0199 × 10^−11^	2.7719 × 10^−1^	3.0199 × 10^−11^	3.0199 × 10^−11^	5.4617 × 10^−9^	3.0199 × 10^−11^	3.3384 × 10^−11^	3.0199 × 10^−11^
F3	3.0199 × 10^−11^	3.0199 × 10^−11^	3.0199 × 10^−11^	3.0199 × 10^−11^	2.4157 × 10^−2^	3.0199 × 10^−11^	3.0199 × 10^−11^	3.0199 × 10^−11^	3.0199 × 10^−11^	2.1544 × 10^−10^	3.0199 × 10^−11^
F4	3.0199 × 10^−11^	3.0199 × 10^−11^	6.7650 × 10^−5^	1.6955 × 10^−2^	8.1527 × 10^−11^	4.5043 × 10^−11^	3.0199 × 10^−11^	3.0199 × 10^−11^	1.2023 × 10^−8^	7.3891 × 10^−11^	3.0199 × 10^−11^
F5	7.2208 × 10^−6^	3.6897 × 10^−11^	8.9934 × 10^−11^	3.0199 × 10^−11^	2.1566 × 10^−3^	3.6897 × 10^−11^	3.0199 × 10^−11^	1.0233 × 10^−1^	4.4205 × 10^−6^	7.2446 × 10^−2^	2.1544 × 10^−10^
F6	6.1001 × 10^−1^	3.0199 × 10^−11^	3.0199 × 10^−11^	3.0199 × 10^−11^	5.1060 × 10^−1^	3.0199 × 10^−11^	6.5277 × 10^−8^	3.3386 × 10^−3^	6.0658 × 10^−11^	8.5000 × 10^−2^	2.1544 × 10^−10^
F7	3.6897 × 10^−11^	3.0199 × 10^−11^	7.0881 × 10^−8^	4.1127 × 10^−7^	4.2067 × 10^−2^	3.0199 × 10^−11^	1.0937 × 10^−10^	2.8129 × 10^−2^	6.0658 × 10^−11^	6.2828 × 10^−6^	3.0199 × 10^−11^
F8	9.2113 × 10^−5^	7.7725 × 10^−9^	3.0199 × 10^−11^	3.0199 × 10^−11^	8.7663 × 10^−1^	6.7220 × 10^−10^	3.0199 × 10^−11^	6.1001 × 10^−1^	2.3897 × 10^−8^	3.9527 × 10^−1^	3.0199 × 10^−11^
F9	3.4742 × 10^−10^	3.0199 × 10^−11^	3.0199 × 10^−11^	5.4941 × 10^−11^	5.9428 × 10^−2^	6.5277 × 10^−8^	3.0199 × 10^−11^	4.0354 × 10^−1^	4.1825 × 10^−9^	3.3679 × 10^−4^	3.0199 × 10^−11^
F10	6.6689 × 10^−3^	3.0199 × 10^−11^	4.1178 × 10^−6^	3.0199 × 10^−11^	1.2023 × 10^−8^	1.9579 × 10^−1^	3.0199 × 10^−11^	5.1857 × 10^−7^	5.4941 × 10^−11^	9.0632 × 10^−8^	8.1014 × 10^−10^
F11	1.6132 × 10^−10^	3.3242 × 10^−6^	3.0199 × 10^−11^	3.0199 × 10^−11^	3.6897 × 10^−11^	2.2360 × 10^−2^	3.0199 × 10^−11^	1.2870 × 10^−9^	5.0723 × 10^−10^	3.0199 × 10^−11^	3.0199 × 10^−11^
F12	1.7769 × 10^−10^	9.2603 × 10^−9^	6.1210 × 10^−10^	3.0199 × 10^−11^	3.0199 × 10^−11^	1.4412 × 10^−2^	3.0199 × 10^−11^	3.0199 × 10^−11^	4.4205 × 10^−6^	3.3384 × 10^−11^	3.0199 × 10^−11^
F13	3.3384 × 10^−11^	9.8329 × 10^−8^	4.4592 × 10^−4^	3.0199 × 10^−11^	3.0199 × 10^−11^	1.4532 × 10^−1^	3.0199 × 10^−11^	3.0199 × 10^−11^	3.0199 × 10^−11^	3.0199 × 10^−11^	3.0199 × 10^−11^
F14	3.0199 × 10^−11^	3.0199 × 10^−11^	3.0199 × 10^−11^	3.0199 × 10^−11^	3.0199 × 10^−11^	3.0199 × 10^−11^	1.1058 × 10^−4^	3.0199 × 10^−11^	3.0199 × 10^−11^	3.0199 × 10^−11^	3.0199 × 10^−11^
F15	5.9428 × 10^−2^	3.1466 × 10^−2^	1.0105 × 10^−8^	7.9782 × 10^−2^	3.5547 × 10^−1^	3.0199 × 10^−11^	3.5638 × 10^−4^	2.3800 × 10^−3^	3.0199 × 10^−11^	1.5846 × 10^−4^	3.0199 × 10^−11^
F16	3.5708 × 10^−6^	1.0666 × 10^−7^	5.8737 × 10^−4^	3.0199 × 10^−11^	3.0199 × 10^−11^	5.4941 × 10^−11^	3.0199 × 10^−11^	1.0702 × 10^−9^	1.6132 × 10^−10^	9.7555 × 10^−10^	8.8910 × 10^−10^
F17	2.4386 × 10^−9^	3.8053 × 10^−7^	3.0939 × 10^−6^	3.0199 × 10^−11^	3.0199 × 10^−11^	1.8500 × 10^−8^	6.5261 × 10^−7^	3.0199 × 10^−11^	3.0199 × 10^−11^	3.0811 × 10^−8^	3.0199 × 10^−11^
F18	3.0199 × 10^−11^	3.0199 × 10^−11^	3.0199 × 10^−11^	3.0199 × 10^−11^	3.0199 × 10^−11^	3.0199 × 10^−11^	4.6159 × 10^−10^	2.8314 × 10^−8^	3.0199 × 10^−11^	3.0199 × 10^−11^	3.0199 × 10^−11^
F19	5.0922 × 10^−8^	6.7650 × 10^−5^	6.1210 × 10^−10^	2.3897 × 10^−8^	8.8411 × 10^−7^	2.1566 × 10^−3^	7.9590 × 10^−3^	2.1540 × 10^−6^	7.3891 × 10^−11^	1.1674 × 10^−5^	3.0199 × 10^−11^
F20	3.8202 × 10^−10^	4.6159 × 10^−10^	1.9527 × 10^−3^	3.0199 × 10^−11^	2.6015 × 10^−8^	1.3111 × 10^−8^	1.0666 × 10^−7^	5.4941 × 10^−11^	3.0199 × 10^−11^	1.6947 × 10^−9^	4.5043 × 10^−11^
F21	5.0912 × 10^−6^	2.4386 × 10^−9^	6.6955 × 10^−11^	3.0199 × 10^−11^	4.5530 × 10^−1^	2.3715 × 10^−10^	3.0199 × 10^−11^	8.1200 × 10^−4^	3.0199 × 10^−11^	8.7663 × 10^−1^	3.0199 × 10^−11^
F22	8.1975 × 10^−7^	3.0199 × 10^−11^	7.1186 × 10^−9^	3.0199 × 10^−11^	5.5329 × 10^−8^	4.7138 × 10^−4^	3.0199 × 10^−11^	2.1540 × 10^−6^	1.8567 × 10^−9^	9.5139 × 10^−6^	6.7220 × 10^−10^
F23	2.8716 × 10^−10^	3.0199 × 10^−11^	7.1186 × 10^−9^	9.5139 × 10^−6^	2.5974 × 10^−5^	3.6897 × 10^−11^	4.9752 × 10^−11^	7.7272 × 10^−2^	3.0199 × 10^−11^	3.7904 × 10^−1^	1.2057 × 10^−10^
F24	9.2603 × 10^−9^	8.1014 × 10^−10^	3.6897 × 10^−11^	7.2884 × 10^−3^	5.7929 × 10^−1^	1.3289 × 10^−10^	1.0937 × 10^−10^	4.8252 × 10^−1^	3.0199 × 10^−11^	9.4696 × 10^−1^	1.0937 × 10^−10^
F25	3.0199 × 10^−11^	3.0199 × 10^−11^	2.8378 × 10^−1^	8.6499 × 10^−1^	3.0199 × 10^−11^	3.0199 × 10^−11^	3.0199 × 10^−11^	3.0199 × 10^−11^	5.5727 × 10^−10^	3.0199 × 10^−11^	3.0199 × 10^−11^
F26	3.9881 × 10^−4^	5.5611 × 10^−4^	9.7555 × 10^−10^	3.0199 × 10^−11^	1.2362 × 10^−3^	1.3703 × 10^−3^	3.0199 × 10^−11^	1.0666 × 10^−7^	4.9752 × 10^−11^	7.7272 × 10^−2^	3.0199 × 10^−11^
F27	6.7650 × 10^−5^	3.0199 × 10^−11^	3.0199 × 10^−11^	3.0199 × 10^−11^	3.0199 × 10^−11^	3.0199 × 10^−11^	3.0199 × 10^−11^	3.0199 × 10^−11^	3.0199 × 10^−11^	3.0199 × 10^−11^	3.0199 × 10^−11^
F28	3.9881 × 10^−4^	3.0199 × 10^−11^	3.0199 × 10^−11^	3.0199 × 10^−11^	8.4848 × 10^−9^	3.0199 × 10^−11^	3.0199 × 10^−11^	1.0000	3.0199 × 10^−11^	1.0666 × 10^−7^	3.0199 × 10^−11^
F29	3.1573 × 10^−5^	4.7445 × 10^−6^	6.9125 × 10^−4^	3.0199 × 10^−11^	2.5306 × 10^−4^	6.7350 × 10^−1^	3.0199 × 10^−11^	1.6238 × 10^−1^	1.0937 × 10^−10^	2.6806 × 10^−4^	3.0199 × 10^−11^
F30	3.0199 × 10^−11^	3.0199 × 10^−11^	3.0199 × 10^−11^	3.0199 × 10^−11^	3.0199 × 10^−11^	3.0199 × 10^−11^	3.0199 × 10^−11^	3.0199 × 10^−11^	3.0199 × 10^−11^	3.0199 × 10^−11^	3.0199 × 10^−11^
